# Strong Selectional Forces Fine-Tune CpG Content in Genes Involved in Neurological Disorders as Revealed by Codon Usage Patterns

**DOI:** 10.3389/fnins.2022.887929

**Published:** 2022-06-10

**Authors:** Rekha Khandia, Anushri Sharma, Taha Alqahtani, Ali M. Alqahtani, Yahya I. Asiri, Saud Alqahtani, Ahmed M. Alharbi, Mohammad Amjad Kamal

**Affiliations:** ^1^Department of Biochemistry and Genetics, Barkatullah University, Bhopal, India; ^2^Department of Pharmacology, College of Pharmacy, King Khalid University, Abha, Saudi Arabia; ^3^Department of Clinical Laboratory Sciences, College of Applied Medical Sciences, University of Hail, Hail, Saudi Arabia; ^4^Institutes for Systems Genetics, Frontiers Science Center for Disease-Related Molecular Network, West China Hospital, Sichuan University, Chengdu, China; ^5^King Fahd Medical Research Center, King Abdulaziz University, Jeddah, Saudi Arabia; ^6^Department of Pharmacy, Faculty of Allied Health Sciences, Daffodil International University, Dhaka, Bangladesh; ^7^Enzymoics, Novel Global Community Educational Foundation, Hebersham, NSW, Australia

**Keywords:** neurodegeneration, metabolism-related genes, codon usage, dinucleotide ratio, RSCU, fine tuning of CpG dinucleotide

## Abstract

Neurodegenerative disorders cause irreversible damage to the neurons and adversely affect the quality of life. Protein misfolding and their aggregation in specific parts of the brain, mitochondrial dysfunction, calcium load, proteolytic stress, and oxidative stress are among the causes of neurodegenerative disorders. In addition, altered metabolism has been associated with neurodegeneration as evidenced by reductions in glutamine and alanine in transient global amnesia patients, higher homocysteine-cysteine disulfide, and lower methionine decline in serum urea have been observed in Alzheimer’s disease patients. Neurodegeneration thus appears to be a culmination of altered metabolism. The study’s objective is to analyze various attributes like composition, physical properties of the protein, and factors like selectional and mutational forces, influencing codon usage preferences in a panel of genes involved directly or indirectly in metabolism and contributing to neurodegeneration. Various parameters, including gene composition, dinucleotide analysis, Relative synonymous codon usage (RSCU), Codon adaptation index (CAI), neutrality and parity plots, and different protein indices, were computed and analyzed to determine the codon usage pattern and factors affecting it. The correlation of intrinsic protein properties such as the grand average of hydropathicity index (GRAVY), isoelectric point, hydrophobicity, and acidic, basic, and neutral amino acid content has been found to influence codon usage. In genes up to 800 amino acids long, the GC3 content was highly variable, while GC12 content was relatively constant. An optimum CpG content is present in genes to maintain a high expression level as required for genes involved in metabolism. Also observed was a low codon usage bias with a higher protein expression level. Compositional parameters and nucleotides at the second position of codons played essential roles in explaining the extent of bias. Overall analysis indicated that the dominance of selection pressure and compositional constraints and mutational forces shape codon usage.

## Introduction

Neurodegenerative disorders are incurable and debilitating pathological conditions resulting in progressive degeneration and possible death of nerve cells. Such diseases pose a major threat to human health due to deterioration in the quality of life and premature mortality. Economic impacts also have been associated with long-term in-home caregiving. Many neurodegenerative disorders have shown an association with misfolding of proteins and their aggregation in specific brain regions (Soto, 2003). The most common neurodegenerative disorders associated with misfolded protein aggregation are Alzheimer’s disease (AD) and Parkinson’s disease (PD). Multiple lines of evidence have connected the link of Aβ and tau in AD and α-syn proteins in PD. However, it is still unclear whether the presence of abnormal proteins is the consequence of disease or its cause (Bourdenx et al., 2017). The presence of misfolded proteins and their aggregation might be attributed to the genetic mutations in genes related to the disease. Other shared pathologies between neurodegenerative diseases are mitochondrial dysfunction, glutamate toxicity, calcium load, proteolytic stress, and oxidative stress (Muddapu et al., 2020). The changes in disease-specific proteins are associated with enhanced oxidative stress, initiation of inflammatory processes, and neuronal damage (Ballard et al., 2011).

A comparative study of genome-wide gene expression data of 93 brain tissue samples obtained from patients with AD, PD, Huntington’s disease (HD), acute myeloid leukemia (AML), and multiple sclerosis revealed a high number of dysregulated genes is associated with each disorder. Still, no gene was shared across all conditions (Durrenberger et al., 2015). This finding indicates that no single shared mechanism is involved in neurodegenerative disorders. However, the results of Durrenberger et al. (2015) did not include an assessment of protein expression and post-translational modifications, which may result in misleading conclusions.

In neurodegenerative diseases, specific neuronal clusters have been found more likely to serve as the primary site for the spread of neuronal pathology (Fu et al., 2018). This vulnerable population exhibits specific morphological features, including long-range neuronal projections and extensive synaptic connections, making them selectively vulnerable due to the higher metabolic requirements for structural integrity maintenance (Pacelli et al., 2015). Muddapu et al. (2020) proposed that the pathological markers of neurodegenerative diseases, including protein misfolding, oxidative stress, and mitochondrial dysfunction, are the direct consequences of metabolic anomalies. For instance, insulin plays a role in cholesterol metabolism essential to myelination and the regulation of amyloid protein degrading enzymes (Wang et al., 2014). Insulin resistance causes an imbalance of glucose metabolism and results in hyperglycemia and oxidative stress, leading to inflammatory response and neuronal damage. Alerted levels of amino acid in the brain and serum of AD patients have been documented. Since glutamate and its metabolite gamma-aminobutyric acid (GABA) are excitatory and inhibitory neurotransmitters, respectively, we can speculate that the alterations in glutamate may adversely affect neural functioning (Esposito et al., 2013). Glutamine and alanine levels are also reduced in the blood of patients with transient global amnesia (Sancesario et al., 2013). Higher homocysteine-cysteine disulfide and lower methionine levels have been documented in the serum of AD patients. In the normal human brain, the activity of the enzyme ornithine transcarbamoylase is very low, thereby preventing the urea cycle (Bensemain et al., 2009). AD patients experience a 44% decline of urea in serum (González-Domínguez et al., 2015). All this evidence suggests the central role of metabolism malfunctioning in neurodegenerative disorders. After observing a potential connection between metabolic disturbances and neurodegeneration, we were tempted to study those metabolism-associated genes that contribute to neurodegeneration if malfunctioning. In case of clinical features associated with neurological consequences like cerebral edema, cerebellar ataxia, coma, seizures, stroke and intellectual disability along with hyperammonemia, protein avoidance, low plasma citrulline and hypoargininemia, commercially, the genetic diagnosis is available, and information regarding the genes those are involved may be obtained. Therefore, we used the information available through commercial sources and assessed a panel of 60 genes associated with neurodegeneration that are directly or indirectly involved in the metabolism or transport of metabolites in brain cells. These genes are associated with several neurodegeneration symptoms, including neurocognitive deficiencies, attention-deficit/hyperactivity disorder, developmental delays, seizures, learning disabilities, lethargy, somnolence, refusal to feed, vomiting, tachypnea, respiratory alkalosis, fatal neonatal encephalopathy with hypotonia, and many others.

All proteins are made up of amino acids, and 61 codons encode 20 amino acids. All amino acids are coded by two or more synonymous codons, excluding methionine and tryptophan. This usage of synonymous codons is not equal, and often, some of the synonymous codons are used preferably over others. This phenomenon is called codon usage bias (CUB), which is attributed to various factors, including overall compositional constraints (Deka and Chakraborty, 2014), selectional or mutational forces (Hershberg and Petrov, 2008), gene expression levels (Zhou et al., 2016), and the tRNA pool (Quax et al., 2015). The gene expression is affected by codon usage choice. The genes with higher expression levels exhibit a higher codon adaptation index (CAI), and the most abundant proteins have higher CAI values (Henry and Sharp, 2007).

Bioinformatics and biomedical research have permitted an expanded understanding of the pathobiology of neurodegenerative disorders. Thus far, little research has been conducted on the genes involved in neurodegeneration from the metabolism perspective. In the present study, the codon usage pattern of 60 relevant genes is studied to elucidate various forces (such as mutational, selectional, or compositional) acting upon them. In the present study, we calculated various indices, including parity and nucleotide skews, to determine the compositional disproportion. Neutrality, parity, ENc-GC3 curve and regression analysis between nucleotide compositions were carried out to reveal the impact of evolutionary forces. In addition, the CAI and relative synonymous codon usage (RSCU) were determined to evaluate the codon preferences. Various statistical methods have been employed to see the association between various molecular features. The analyses helped determine various molecular signatures, evolutionary forces acting on genes and codon usage patterns related to the genes involved in metabolism and neurodegeneration. The results of this study will provide insight into the factors affecting codon choices along with the expression level information of these genes.

## Materials and Methods

### Data Collection

The genes analyzed for neurodegenerative disorders were obtained from the NCBI Genetic Testing Registry NGS Neurodegenerative disorders Multi-Gene Panel. For neurodegenerative symptoms with evidence of disturbed metabolism (cerebral edema, cerebellar ataxia, coma, seizures, stroke, and intellectual disability along with hyperammonemia, protein avoidance, low plasma citrulline and hypoargininemia), next-generation sequencing is recommended. Laboratory of genome diagnostics, LGD-AUMC Academic Medical Center, University of Amsterdam, offers NGS for a Multi-Gene Panel for diagnostic purposes. The gene panel offered by them was taken in the present study. A total of 183 transcripts belonging to 60 genes were studied, shown in Table 1 with the function of each gene and the number of its transcripts utilized. To be utilized in the study, a transcript/coding sequence (CDS) must be in a reading frame and contain no nucleotides other than A, T, G, or C (for example, R, Y, or B representing A/G, C/T, and C/G/T respectively). In addition, the sequences were devoid of UAA, UAG, or UGA stop codons within sequences. A total of 264096 nucleotides and 88032 codons were studied.

**TABLE 1:** The list of genes involved in neurodegenerative disorders with the location on the human chromosome, the disease involved, and the number of transcripts.

S No.	Name of gene	Synonym	Location of chromosome	Disease involved	Neurological manifestations	No. of transcript studied	Metabolic function (if any)
1	*ABCD1*	*ALD; AMN; ALDP; ABC42*	Xq28	Adrenoleukodystrophy	X-linked adrenoleukodystrophy (X-ALD) affects the white matter and the adrenal cortex.	1	Encodes a transporter protein localized into the peroxisomal membrane and participate in the metabolism of very-long-chain fatty acids
2	*ADSL*	*ASL; AMPS; ASASE*	22q13.1	Adenylosuccinate lyase deficiency	A disorder characterized by intellectual disability, psychomotor delay and/or regression, seizures, and autistic features	4	Participate in biosynthesis of purines
3	*ALDH7A1*	*EPD; PDE; ATQ1*	5q23.2	Pyridoxine dependent epilepsy	Intractable seizures within the first weeks to months of life. Response obtained to large daily supplements of pyridoxine	2	Codes for enzyme α-aminoadipic semialdehyde (α-AASA) dehydrogenase that breakdown lysine in the brain
4	*APTX*	*AOA; AOA1; AXA1; EAOH; EOAHA; FHA-HIT*	9p21.1	Coenzyme Q10 deficiency	Associated with multisystem involvement, including neurologic manifestations such as fatal neonatal encephalopathy with hypotonia; a late-onset of slowly progressive multiple-system atrophy-like phenotype (neurodegeneration with autonomic failure and various combinations of parkinsonism and cerebellar ataxia, and pyramidal dysfunction); and dystonia, spasticity, seizures, and intellectual disability.	23	Role in single stranded DNA repair
5	*ARG1*	*arginase1*	6q23.2	Arginase deficiency	Arginase deficiency in untreated individuals is characterized by episodic hyperammonaemia of variable degree that is infrequently severe enough to be life threatening or to cause death.	3	A manganese-containing enzyme catalyzing the final step in the urea cycle for disposal of toxic ammonia by converting l-arginine to l-ornithine and urea
6	*ARSA*	*ASA; MLD*	22q13.33	Metachromatic leukodystrophy	Arylsulfatase A deficiency (also known as metachromatic leukodystrophy or MLD) is characterized by characterized by the damage of the myelin sheath resulting in progressive motor and cognitive impairment as clinical manifestations	6	Responsible for coding the enzyme arylsulfatase A that help in processing sulfatides a subgroup of sphingolipids
7	*ASL*	*ASAL*	7q11.21	Arginosuccinate lyase deficiency	The severe neonatal-onset form is characterized by hyperammonaemia (Neurocognitive deficiencies, attention-deficit/hyperactivity disorder, developmental delay, seizures, and learning disability) within the first few days after birth that can manifest as increasing lethargy, somnolence, refusal to feed, vomiting, tachypnea, and respiratory alkalosis. Absence of treatment leads to worsening lethargy, seizures, coma, and even death	4	Involved in Alanine, aspartate and glutamate metabolism, urea cycle metabolism and metabolism of amino acids.
8	*ASS1*	*ASS; CTLN1*	9q34.11	Citrullinemia type I	Later-onset form is associated with intense headaches, blind spots (scotomas), problems with balance and muscle coordination (ataxia), and lethargy.	2	Codes for argininosuccinate synthase 1 enzyme. It participates in the urea cycle
9	*BCKDHA*	*MSU; MSUD1; OVD1A; BCKDE1A*	19q13.2	Maple syrup urine disease	Accumulation of leucine, isoleucine, and valine and their byproducts is toxic to the nervous system and lead to seizures, developmental delay, and the other health problems associated with maple syrup urine disease.	2	Encodes for alpha-keto acid dehydrogenase involved in breakdown of leucine, isoleucine, and valine
10	*BCKDHB*	*E1B; BCKDE1B; BCKDH E1-beta*	6q14.1	Maple syrup urine disease	Accumulation of leucine, isoleucine, and valine and their byproducts is toxic to the nervous system and lead to seizures, developmental delay, and the other health problems associated with maple syrup urine disease.	3	Encodes for alpha-keto acid dehydrogenase involved in breakdown of leucine, isoleucine, and valine
11	*CBS*	*CBSL; HIP4*	21q22.3	Classic homocystinuria	Characterized by involvement of the eye, skeletal system, vascular system and CNS	5	Codes for cystathionine beta-synthase which converts homocysteine and serine to cytathionine
12	*COQ2*	*MSA1; CL640; COQ10D1; PHB:PPT*	4q21.23	Coenzyme Q10 deficiency, primary 1	Primary coenzyme Q10 (CoQ10) deficiency is usually associated with multisystem involvement, including neurologic manifestations such as fatal neonatal encephalopathy with hypotonia; a late-onset of slowly progressive multiple-system atrophy-like phenotype (neurodegeneration with autonomic failure and various combinations of parkinsonism and cerebellar ataxia, and pyramidal dysfunction); and dystonia, spasticity, seizures, and intellectual disability.	2	Required in the biosynthetic pathway of COQ (ubiquinone). This enzyme catalyzes the prenylation of p-hydroxybenzoate with an all-trans polyprenyl group.
13	*COQ8A*	*COQ8; ADCK3; ARCA2; CABC1; SCAR9; COQ10D4*	1q42.13	Coenzyme Q10 deficiency, primary 1	Deficiency is usually associated with multisystem involvement, including neurologic manifestations such as fatal neonatal encephalopathy with hypotonia; a late-onset slowly progressive multiple-system atrophy-like phenotype (neurodegeneration with autonomic failure and various combinations of parkinsonism and cerebellar ataxia, and pyramidal dysfunction); and dystonia, spasticity, seizures, and intellectual disability.	1	Required in the biosynthetic pathway of CoQ (ubiquinone). This enzyme catalyzes the prenylation of p-hydroxybenzoate with an all-trans polyprenyl group
14	*COQ9*	*COQ10D5; C16orf49*	16q21	Coenzyme Q10 deficiency, primary 1	Primary coenzyme Q10 (CoQ10) deficiency is usually associated with multisystem involvement, including neurologic manifestations such as fatal neonatal encephalopathy with hypotonia; a late-onset slowly progressive multiple-system atrophy-like phenotype (neurodegeneration with autonomic failure and various combinations of parkinsonism and cerebellar ataxia, and pyramidal dysfunction); and dystonia, spasticity, seizures, and intellectual disability.	1	Required in the biosynthetic pathway of CoQ (ubiquinone). This enzyme catalyzes the prenylation of p-hydroxybenzoate with an all-trans polyenyl group
15	*CPS1*	*PHN; GATD6; CPSASE1*	2q34	Congenital hyperammonemia, type I	A rare, severe disorder of urea cycle metabolism typically characterized by either a neonatal-onset of severe hyperammonemia that occurs few days after birth and manifests with lethargy, vomiting, hypothermia, seizures, coma and death or a presentation outside the new-born period at any age with (sometimes) milder symptoms of hyperammonemia	4	Encodes for carbamoyl phosphate synthetase I. It converts ammonium into carbamoyl phosphate, and plays an intricate role in arginine metabolism and pyrimidine metabolism
16	*CYP27A1*	*CTX; CP27; CYP27*	2q35	Cerebrotendinous xanthomatosis (Cholestanol storage disease)	Cerebrotendinous xanthomatosis (CTX) is a lipid storage disease characterized by infantile-onset diarrhea, childhood-onset cataract, adolescent- to young adult-onset tendon xanthomas, and adult-onset progressive neurologic dysfunction (dementia, psychiatric disturbances, pyramidal and/or cerebellar signs, dystonia, atypical parkinsonism, peripheral neuropathy, and seizures).	1	Encodes for sterol 27-hydroxylase. It breaks down cholesterol to form a bile acid called chenodeoxycholic acid. Maintains normal cholesterol levels in the body.
17	*DBT*	*E2; E2B; BCATE2; BCKADE2; BCKAD-E2; BCKDH-E2; BCOADC-E2*	1p21.2	Maple syrup urine disease	Elevated concentrations of branched-chain amino acids (BCAAs; leucine, isoleucine, and valine) and alloisoleucine, as well as a generalized disturbance of amino acid concentration ratios, are present in blood and the maple syrup odor can be detected in crewmen	1	Encodes for a part of branched-chain alpha-keto acid dehydrogenase (BCKD) enzyme complex. It helps in the breakdown of the branched amino acids leucine, isoleucine, and valine
18	*DDC*	*AADC*	7p12.2-12.1	Deficiency of aromatic-L-amino-acid decarboxylase	Autosomal recessive inborn error in neurotransmitter metabolism that leads to combined serotonin and catecholamine deficiency	7	Encodes for aromatic l-amino acid decarboxylase (AADC) enzyme. It converts L-dopa and 5-hydroxytryptophan to dopamine and serotonin
19	*DLD*	*LAD; DLDD; DLDH; GCSL; PHE3; OGDC-E3*	7q31.1	Maple syrup urine disease	Characterized by an overlapping continuum that ranges from early-onset neurologic manifestations to adult-onset liver involvement and, rarely, a myopathic presentation.	4	Encodes for an enzyme called dihydrolipoamide dehydrogenase. Involved in metabolism of leucine, isoleucine, and valine
20	*GAMT*	*PIG2; CCDS2; TP53I2; HEL-S-20*	19p13.3	Inborn errors of creatine metabolism	GAMT deficiency is characterized by Symptoms ranging from mild intellectual disability and speech delay to severe intellectual disability, seizures, movement disorder, and behavior disorder	2	This enzyme participates in glycine, serine and threonine metabolism and arginine and proline metabolism.
21	*GATM*	*AGAT; CCDS3; FRTS1*	15q21.1	Inborn errors of creatine metabolism	Intellectual disability and seizures, behavior disorder that can include autistic behaviors and self-mutilation	2	Encodes for arginine:glycine amidinotransferase. Participate in synthesis of glycine, arginine, and methionine.
22	*GCDH*	*GCD; ACAD5*	19p13.13	Glutaric aciduria, type 1	Result in acute bilateral striatal injury and subsequent complex movement disorders. increased risk for renal disease	2	Encodes for glutaryl-CoA dehydrogenase. Involved in the metabolism of tryptophan, lysine and hydroxylysine
23	*GCH1*	*GCH; DYT5; DYT14; DYT5a; GTPCH1; HPABH4B; GTP-CH-1*	14q22.2	Dopa-responsive dystonia (Segawa syndrome)	Typically characterized by signs of parkinsonism that may be relatively subtle. Such signs may include slowness of movement (bradykinesia), tremors, stiffness and resistance to movement (rigidity), balance difficulties, and postural instability.	4	Encodes for GTP cyclohydrolase 1. Involved in the production of a molecule called tetrahydrobiopterin, that process phenylalanine into tyrosine
24	*GNS*	*G6S*	12q14.3	Sanfilippo syndrome	A rare autosomal recessive lysosomal storage disease affecting the metabolism of mucopolysaccharides. Signs and symptoms include behavioral changes, sleep disorders, mental developmental delays, and seizures	1	Affect the Metabolism of mucopolysaccharides. Some related pathways Glycosaminoglycan metabolism is other related pathway
25	*HCLS*	*HCS*	21q22.13	Holocarboxylase synthetase deficiency	Deficiency leads to complications of metabolic acidosis, seizure, and hyperammonemia that can result in long-term neurological sequelae and developmental disability	8	Encodes for Holocarboxylase synthetase. Related to inborn error of biotin metabolism
26	*HGSNAT*	*RP73; HGNAT; MPS3C; TMEM76*	8p11.21-11.1	Sanfilippo syndrome	Signs and symptoms include behavioral changes, sleep disorders, mental developmental delays, and seizures	4	This gene encodes a lysosomal acetyltransferase, which is one of several enzymes involved in the lysosomal degradation of heparin sulfate
27	*HPRT1*	*HPRT; HGPRT*	Xq26.2-26.3	Lesch-Nyhan syndrome	Lesch-Nyhan disease (LND) at the most severe end with motor dysfunction resembling severe cerebral palsy, intellectual disability, and self-injurious behavior	1	Encodes for hypoxanthine phosphoribosyltransferase 1. Rare disorder of purine metabolism
28	*IDS*	*ID2S; MPS2; SIDS*	Xq28	Mucopolysaccharidosis Type I/II	Short stature; macrocephaly with or without communicating hydrocephalus; macroglossia; hoarse voice; conductive and sensorineural hearing loss; hepatosplenomegaly; dysostosis multiplex; spinal stenosis; and carpal tunnel syndrome.	2	Codes for iduronate 2-sulfatase responsible for breakdown of large sugar molecules glycosaminoglycans
29	*IDUA*	*IDA; MPS1; MPSI*	4p16.3	Mucopolysaccharidosis Type I/II	Neurological complications may include damage to neurons. Pain and impaired motor function (ability to start and control muscle movement) may result from compressed nerves or nerve roots in the spinal cord or in the peripheral nervous system.	3	Encodes for alpha-L-iduronidase which is an enzyme involved in the metabolism of glycosaminoglycans (GAGs).
30	*IVD*	*IVDH; ACAD2*	15q15.1	Isovaleryl-CoA dehydrogenase deficiency	A rare autosomal disorder. It is characterized by abnormalities in the metabolism of leucine. The genetic deficiency of IVD results in an accumulation of isovaleric acid, which is toxic to the central nervous system and leads to isovaleric acidemia	7	Encodes for Isovaleryl-CoA dehydrogenase, a mitochondrial matrix enzyme that catalyzes the third step in leucine catabolism.
31	*LMBRD1*	*NESI; LMBD1; MAHCF; C6orf209*	6q13	Cobalamin F disorder	Cognitive and neurological impairment	4	Encodes for LMBR1 domain containing 1, involved in transport and metabolism of cobalamin
32	*MAN2B1*	*MANB; LAMAN*	19p13.13	Methylmalonic acidemia with homocystinuria	Developmental delay, eye defects, neurological problems, and blood abnormalities.	2	Encoding for alpha-mannosidase. Non-functional LMBD1 protein prevents the release of vitamin B12 from lysosomes. Ultimately decrease in the production of methionine and accumulation of homocysteine
33	*MILYCD*	*MCD*	16q23.3	Malonic Aciduria	Developmental delay in early childhood, seizures, hypotonia, diarrhea, vomiting, metabolic acidosis, hypoglycemia, ketosis, abnormal urinary compounds, lactic acidemia, and hypertrophic cardiomyopathy	1	Encodes for malonyl-CoA decarboxylase involved in the metabolism of fatty acids synthesis and is important in muscle and brain metabolism
34	*MMAA*	*cblA*	4q31.21	Vitamin B12-responsive methylmalonic acidemia type cblA	In the neonatal period the disease can present with lethargy, vomiting, hypotonia, hypothermia, respiratory distress, severe ketoacidosis, hyperammonemia, neutropenia, and thrombocytopenia and can result in death within the first four weeks of life. In the infantile/non-B12-responsive phenotype, infants are normal at birth, but develop lethargy, vomiting, dehydration, failure to thrive, hepatomegaly, hypotonia, and encephalopathy within a few weeks to months of age. Major secondary complications of methylmalonic acidemia include: intellectual impairment (variable); tubulointerstitial nephritis with progressive renal failure; “metabolic stroke”	2	Encodes for product involved in certain proteins, fats (lipids), and cholesterol and transport of vitamin B12
35	*MMAB*	*cblB*	12q24.11	Vitamin B12-responsive methylmalonic acidemia type cblB	Major secondary complications of methylmalonic acidemia includes intellectual impairment (variable); tubulointerstitial nephritis with progressive renal failure; “metabolic stroke” (acute and chronic basal ganglia injury) causing a disabling movement disorder with choreoathetosis, dystonia, and para/quadriparesis; pancreatitis; growth failure; functional immune impairment; and optic nerve atrophy.	1	Metabolism of cobalamin associated B
36	*MMACHC*	*cblC*	1p34.1	Methylmalonic acidemia with homocystinuria	In Adolescents and adults, may present neuropsychiatric symptoms, progressive cognitive decline, thromboembolic complications, and/or sub-acute combined degeneration of the spinal cord.	2	Encoded enzyme helps in converting vitamin B12 into adenosylcobalamin (AdoCbl) or methylcobalamin.
37	*MMADHC*	*cblD; C2orf25; CL25022*	2q23.2	Homocystinuria, Methylmalonic acidemia, Methylmalonic acidemia with homocystinuria	Infants may present with poor feeding and slow growth, neurologic abnormality, and, rarely, hemolytic uremic syndrome (HUS).	1	Encodes for a protein involved in converting vitamin B12 into adenosylcobalamin (AdoCbl) or methylcobalamin (MeCbl)
38	*MMUT*	*MCM; MUT*	6p12.3	Methylmalonic acidemia	All phenotypes are characterized by periods of relative health and intermittent metabolic decompensation. Major secondary complications of methylmalonic acidemia includes intellectual impairment (variable); tubulointerstitial nephritis with progressive renal failure; “metabolic stroke” (acute and chronic basal ganglia injury) causing a disabling	1	Encodes for Methyl malonyl CoA mutase, responsible for a particular step in the breakdown of several protein building blocks (amino acids), specifically isoleucine, methionine, threonine, and valine. The enzyme also helps break down certain types of fats (lipids) and cholesterol. Among its related pathways are Amino Acid metabolism and Carbon metabolism
39	*MOCS1*	*MIG11; MOCOD; MOCS1A; MOCS1B*	6p21.2	Combined molybdoflavoprotein enzyme deficiency	Molybdenum cofactor deficiency is a rare condition characterized by brain dysfunction (encephalopathy), atrophy of brain tissue, microcephaly	7	Encodes for protein involved in in the formation (biosynthesis) of a molecule called molybdenum cofactor.
40	*MOCS2*	*MPTS; MCBPE; MOCO1; MOCODB*	5q11.2	Combined molybdoflavoprotein enzyme deficiency	Rare autosomal recessive metabolic disorder characterized by neonatal onset of intractable seizures, opisthotonus, and facial dysmorphism associated with hypouricemia and elevated urinary sulfite levels	2	Encodes for molybdopterin synthase involved in formation (biosynthesis) of a molecule called molybdenum cofactor.
41	*MTHFR*	*MTHR_HUMAN*	1p36.22	Homocystinuria due to MTHFR deficiency	A genetic mutation that may lead to high levels of homocysteine in the blood and low levels of folate and other vitamins. Leads to depression, anxiety, bipolar disorder, nerve pain etc.	2	A key enzyme involved in the metabolism of folate and break down of homocysteine.
42	*MTR*	*MS; HMAG; cblG*	1q43	Homocystinuria	Adolescents and adults, may have neuropsychiatric symptoms, progressive cognitive decline, thromboembolic complications, and/or subacute combined degeneration of the spinal cord.	3	Encodes for methionine synthase involved in converting amino acid homocysteine to methionine
43	*MTRR*	*MSR; cblE*	5p15.31	Homocystinuria	ELevated levels of homocysteine results neurodegenerative disorders, recurrent pregnancy loss, neural tube defects	5	Encodes for methionine synthase reductase. Involved in conversion of homocysteine to methionine.
44	*NAGLU*	*NAG; CMT2V; MPS3B; UFHSD; MPS-IIIB*	17q21.2	Charcot-Marie-Tooth disease, Mucopolysaccharidosis type III	A rare autosomal recessive lysosomal storage disease with signs and symptoms of behavioral changes, sleep disorders, mental developmental delays, and seizures	1	Encodes for N-acetyl-alpha-glucosaminidase involved in the step-wise breakdown of large molecules called glycosaminoglycans
45	*NPC1*	*NPC; POGZ; SLC65A1*	18q11.2	Niemann-Pick disease,	Patients with the classical phenotype present progressive neurological disease in late infancy to adolescence, with clumsy gait, ataxia, school failure, behavior problems and a characteristic supranuclear downward gaze paralysis	1	Encodes for a protein present in membranes of lysosomes and endosomes, involved in movement of cholesterol and other types of fats (lipids) within cells and across cell membranes.
46	*NPC2*	*HE1; EDDM1*	14q24.3	Niemann-Pick disease,	Patients with the classical phenotype present progressive neurological disease in late infancy to adolescence, with clumsy gait, ataxia, school failure, behavior problems and a characteristic supranuclear downward gaze paralysis	3	Encodes for a protein present in membranes of lysosomes and endosomes, involved in movement of cholesterol and other types of fats (lipids) within cells and across cell membranes.
47	*OTC*	*OCTD; OTCD*	Xp11.4	Ornithine carbamoyl transferase deficiency	A rare, genetic disorder of urea cycle metabolism and ammonia detoxification. Ornithine transcarbamylase (OTC) deficiency neuropsychological complications include developmental delay, learning disabilities, intellectual disability, attention-deficit/hyperactivity disorder (ADHD), and executive function deficits	1	Urea cycle metabolism and ammonia detoxification
48	*PCBD1*	*PCD; PHS; DCOH; PCBD*	10q22.1	Tetrahydrobiopterin deficiency	Abnormality of the nervous system; Brain and/or spinal cord issue; Neurologic abnormalities; Neurological abnormality	3	Codes for enzyme pterin-4 alpha-carbinolamine dehydratase involved in recycling of tetrahydrobiopterin. Tetrahydrobiopterin helps in converting phenylalanine into another amino acid, tyrosine
49	*PCCA*	*Propionyl-CoA Carboxylase Subunit Alpha*	13q32.3	Propionic acidemia	Developmental delays or regression, movement disorders, or cardiomyopathy.	11	Codes for propionyl-CoA carboxylase subunit alpha that Helps in propionate metabolism
50	*PCCB*	*PCCase Subunit beta*	3q22.3	Propionic acidemia	Growth impairment, intellectual disability, seizures, basal ganglia lesions	2	Encodes for an enzyme propionyl-CoA carboxylase. Helps in breakdown of isoleucine, methionine, threonine, and valine
51	*PDSS1*	*DPS; SPS; TPT; COQ1; TPRT; COQ1A; TPT 1; hDPS1; COQ10D2*	10p12.1	Coenzyme Q10 deficiency	In the most severe cases, the condition becomes apparent in infancy and causes severe brain dysfunction combined with muscle weakness (encephalomyopathy) and the failure of other body systems	3	Involved in CoQ biosynthesis
52	*PDSS2*	*DLP1; COQ1B; hDLP1; COQ10D3; C6orf210; bA59I9.3*	6q21	Coenzyme Q10 deficiency, primary 1	Deficiency is usually associated with multisystem involvement, including neurologic manifestations such as fatal neonatal encephalopathy with hypotonia; a late-onset slowly progressive multiple-system atrophy-like phenotype (neurodegeneration with autonomic failure and various combinations of parkinsonism and cerebellar ataxia, and pyramidal dysfunction); and dystonia, spasticity, seizures, and intellectual disability.	1	Metabolism of coenzymeQ
53	*PNP*	*NP; PUNP; PRO1837*	14q11.2	Purine-nucleoside phosphorylase deficiency	A rare immune disease characterized by progressive immunodeficiency leading to recurrent and opportunistic infections, autoimmunity and malignancy as well as neurologic manifestations	1	Eccodes for Purine-nucleoside phosphorylase. PNP catalyzes the reversible cleavage of inosine to hypoxanthine and guanosine to guanine
54	*PNPO*	*PDXPO; HEL-S-302*	17q21.32	Pyridoxal phosphate-responsive seizures	PNPOD is an autosomal recessive inborn error of metabolism resulting in vitamin B6 deficiency that manifests as neonatal-onset of severe seizures and subsequent encephalopathy.	1	Encodes for pyridoxamine 5′-phosphate oxidase. It is involved in the metabolism of vitamin B6.
55	*PTS*	*PTPS*	11q23.1	BH4-deficient hyperphenylalaninemia A	6-pyruvoyl-tetrahydropterin synthase (PTPS) deficiency is one of the causes of malignant hyperphenylalaninemia due to tetrahydrobiopterin deficiency. Not only does tetrahydrobiopterin deficiency cause hyperphenylalaninemia, it is also responsible for defective neurotransmission of monoamines because of malfunctioning tyrosine and tryptophan hydroxylases	1	Encodes for 6-pyruvoyltetrahydropterin synthase. Indirectly involved in processing of several amino acids
56	*QDPR*	*DHPR; PKU2; HDHPR; SDR33C1*	4p15.32	Dihydropteridine reductase deficiency	An autosomal recessive condition characterized by BH4-defecient hyperphenylalaninemia, depletion of dopamine and serotonin, and progressive cognitive and motor deficits.	2	Encodes for Quinonoid dihydropteridine reductase (QDPR) catalyses the regeneration of tetrahydrobiopterin (BH4), a cofactor for monoamine synthesis, phenylalanine hydroxylation and nitric oxide production
57	*SGSH*	*HSS; SFMD; MPS3A*	17q25.3	Sanfilippo syndrome	A rare autosomal recessive lysosomal storage disease with symptoms of behavioral changes, sleep disorders, mental developmental delays	3	Metabolism of mucopolysaccharide. Glycosaminoglycan metabolism in Lysosome
58	*SLC2A1*	*CSE; PED; DYT9; GLUT; DYT17; DYT18; EIG12; GLUT1; HTLVR; GLUT-1; SDCHCN; GLUT1DS*	1p34.2	GLUT1 deficiency syndrome 1	Classic phenotype is characterized by infantile-onset seizures, delayed neurologic development, acquired microcephaly, and complex movement disorders.	1	Encodes for a protein called protein called the glucose transporter protein type 1 (GLUT1). n the brain it is involved in the movement of glucose across the blood brain barrier. Protect and maintain neurons.
59	*SLC6A19*	*HND; B0AT1*	5p15.33	Hartnup disease	A rare metabolic disorder belonging to the neutral aminoaciduria, mainly characterized by skin photosensitivity, ocular and neuropsychiatric features, due to abnormal renal and gastrointestinal transport of neutral amino acids	1	Codes for protein called system B(0) neutral amino acid transporter 1 (B0AT1). B0AT1 transports the neutral amino acids
60	*SPR*	*SDR38C1*	2p13.2	Dopa-responsive dystonia due to sepiapterin reductase deficiency	Deficiency (SRD), which ranges from significant motor and cognitive deficits to only minimal findings. Clinical features include motor and speech delay, parkinsonian signs, intellectual disability, psychiatric and/or behavioral abnormalities	1	Codes for epiapterin reductase enzyme. Participates indirectly in processing amino acids

### Nucleobase Compositional Analysis

The nucleobase composition was calculated for all the 183 CDSs. The number of A, T, C, G nucleotides present, the % of the nucleotides, and % composition at the first, second, and third codon position (A1, T1, C1, G1, A2, T2, C2, G2, A3, T3, C3, G3) were determined. Total AT% and GC%, along with AT3% and GC3%, were calculated. Calculations of %GC at the first and second place (GC12) and GC% content at the third place were also included. The %GC at different codon positions (%GC1,%GC2, and %GC3) helps decipher the relationship between the codon usage and compositional, selectional, and mutational forces (Mazumder et al., 2014). The above calculations were performed using informatics software developed by Puigbò et al. (2008) and available at http://genomes.urv.es/CAIcal/ (Supplementary Table 1).

### Dinucleotide Abundance

Sixteen dinucleotides obtained from combining four nucleotides were subjected to odds ratio analysis. The odds ratios, the results of dividing the observed frequencies by expected frequencies, were calculated and presented in Table 2. The calculations were performed using DNASTAR Lasergene Inc.^1^ software. The dinucleotides with an odds ratio less than 0.78 are considered underrepresented, and greater than 1.25 are considered overrepresented (Kunec and Osterrieder, 2016).

**TABLE 2:** Dinucleotide analysis showing odds ratio of genes.

	Dinucleotide odds ratio															
Gene name	AA	AC	AG	AT	CA	CC	CG	CT	GA	GC	GG	GT	TA	TC	TG	TT
*ABCD1*	0.429	0.744	1.087	0.486	1.123	1.709	0.994	1.295	0.923	1.731	1.810	0.787	0.272	0.937	1.359	0.315
*ADSL*	0.924	0.913	1.260	1.022	1.345	0.892	0.521	1.045	1.289	1.178	0.995	0.718	0.551	0.821	1.413	1.113
*ALDH7A1*	1.340	0.729	1.329	0.945	1.107	0.740	0.319	1.080	1.334	0.967	1.361	0.924	0.562	0.810	1.577	0.875
*APTX*	1.458	0.796	1.420	0.969	1.265	0.973	0.224	1.028	1.365	0.942	1.237	0.734	0.553	0.777	1.400	0.859
*ARG1*	1.580	1.125	1.377	0.862	1.221	0.820	0.204	1.173	1.455	0.670	1.287	0.670	0.688	0.802	1.215	0.850
*ARSA*	0.259	0.883	0.857	0.421	1.050	2.358	0.721	1.608	0.843	1.619	1.648	0.614	0.270	0.876	1.497	0.477
*ASL*	0.559	0.921	1.280	0.591	1.386	1.345	0.721	1.150	1.177	1.495	1.751	0.703	0.218	0.841	1.386	0.477
*ASS1*	1.099	0.866	1.241	0.788	1.422	1.512	0.659	0.931	1.137	1.099	1.396	0.814	0.323	1.047	1.163	0.504
*BCKDHA*	0.563	0.958	1.168	0.755	1.432	1.557	0.887	1.066	1.006	1.492	1.617	0.635	0.443	0.934	1.078	0.407
*BCKDHB*	1.117	0.659	1.088	1.165	1.083	0.900	0.409	1.054	1.083	0.934	1.237	0.809	0.746	0.953	1.329	1.434
*CBS*	0.667	0.888	1.270	0.619	1.246	1.349	0.983	0.915	1.397	1.427	1.843	0.756	0.135	0.828	1.327	0.350
*COQ2*	0.894	0.847	0.881	0.847	0.941	1.116	0.672	1.277	0.908	1.277	1.405	0.800	0.726	0.766	1.432	1.210
*COQ8A*	0.659	0.791	1.293	0.634	1.326	1.655	0.725	1.169	1.194	1.474	1.556	0.543	0.189	0.955	1.202	0.634
*COQ9*	0.653	0.904	1.490	0.669	1.556	1.255	0.569	1.054	1.205	1.523	1.406	0.720	0.301	0.753	1.389	0.552
*CPS1*	1.363	0.845	1.187	1.110	1.291	0.913	0.195	1.036	1.294	0.785	1.051	0.820	0.553	0.892	1.521	1.143
*CYP27A1*	0.652	0.853	1.194	0.562	1.134	1.585	0.682	1.334	1.113	1.485	1.434	0.702	0.361	0.813	1.424	0.672
*DBT*	1.735	0.807	1.138	1.293	1.116	0.751	0.199	1.116	1.160	0.707	0.707	0.796	0.961	0.917	1.326	1.271
*DDC*	0.980	0.807	1.173	0.879	1.244	1.119	0.540	1.140	1.223	1.282	1.325	0.701	0.381	0.834	1.505	0.865
*DLD*	1.598	0.691	1.313	1.279	1.122	0.437	0.160	0.948	1.301	0.813	0.936	0.902	0.859	0.725	1.544	1.373
*GAMT*	0.485	1.001	0.916	0.600	1.243	1.780	1.074	1.085	1.053	1.580	1.822	0.611	0.221	0.822	1.253	0.453
*GATM*	1.188	0.958	1.017	1.240	1.284	1.054	0.453	1.039	1.240	0.921	0.869	0.661	0.690	0.898	1.351	1.136
*GCDH*	0.553	0.824	1.199	0.664	1.168	1.377	0.879	1.168	1.254	1.433	1.722	0.726	0.258	0.959	1.341	0.473
*GCDH1*	1.034	0.635	1.383	0.793	1.051	1.332	0.967	0.849	1.237	1.529	1.501	0.691	0.517	0.703	1.113	0.663
*GNS*	1.110	1.042	1.119	0.926	1.255	1.090	0.386	1.197	1.139	0.878	1.052	0.820	0.685	0.917	1.341	1.042
*HCLS*	0.982	0.926	1.333	0.759	1.221	1.177	0.495	1.153	1.314	0.883	1.217	0.871	0.482	1.060	1.240	0.886
*HGSNAT*	0.872	0.733	0.877	0.961	0.985	1.052	0.464	1.442	1.057	1.045	1.173	0.824	0.529	1.112	1.586	1.288
*HPRT1*	1.463	0.756	1.049	1.512	0.927	0.610	0.195	0.951	1.463	0.634	0.976	0.829	0.927	0.683	1.683	1.341
*IDS*	0.807	0.905	0.882	1.004	1.149	1.633	0.527	1.252	1.106	0.897	1.008	0.736	0.535	1.126	1.330	1.102
*IDUA*	0.314	1.027	0.827	0.276	1.103	2.185	1.549	1.196	0.823	2.007	1.502	0.679	0.204	0.815	1.133	0.361
*IVD*	0.717	0.701	1.210	0.788	1.158	1.150	0.568	1.321	1.144	1.484	1.556	0.724	0.390	0.862	1.581	0.647
*LMBRD1*	1.531	0.755	0.911	1.454	0.853	0.499	0.203	1.227	1.040	0.628	0.672	0.796	1.227	0.900	1.350	1.954
*MAN2B1*	0.622	1.060	0.973	0.559	1.305	1.561	0.949	1.176	0.986	1.553	1.508	0.696	0.295	0.818	1.319	0.617
*MLYCD*	0.778	0.756	1.124	0.465	1.016	1.275	1.210	1.242	1.080	1.815	1.675	0.659	0.248	0.897	1.221	0.540
*MMAA*	1.605	0.917	1.299	1.045	1.159	0.739	0.242	1.070	1.261	0.713	1.032	0.803	0.841	0.841	1.236	1.197
*MMBB*	0.936	0.851	1.319	0.681	1.191	1.362	0.894	0.979	1.255	1.277	1.511	0.681	0.404	0.936	1.000	0.723
*MMACHC*	0.525	1.039	1.249	0.598	1.217	2.203	0.493	1.374	1.144	1.311	1.112	0.567	0.525	0.734	1.280	0.630
*MMADHC*	1.564	0.845	1.151	1.222	1.079	0.593	0.144	1.097	1.330	0.647	0.755	0.899	0.791	0.827	1.600	1.456
*MMUT*	1.655	0.746	1.187	1.300	1.101	0.675	0.220	1.052	1.286	0.803	0.980	0.718	0.845	0.824	1.400	1.208
*MOCS1*	0.687	0.697	1.391	0.685	1.347	1.555	0.517	1.214	1.154	1.431	1.533	0.672	0.267	0.951	1.354	0.546
*MOCS2*	1.654	0.462	1.519	1.250	1.000	0.788	0.327	0.885	1.365	0.885	0.692	0.942	0.846	0.865	1.365	1.154
*MTHFR*	0.784	0.941	1.315	0.603	1.217	1.591	0.610	1.221	1.366	1.177	1.402	0.677	0.276	0.929	1.296	0.595
*MTR*	1.342	0.818	1.291	1.099	1.155	0.950	0.311	1.021	1.375	0.931	1.139	0.706	0.677	0.738	1.410	1.036
*MTRR*	1.359	0.894	1.296	0.979	1.298	1.006	0.317	1.106	1.160	0.894	0.889	0.738	0.709	0.933	1.181	1.243
*NAGLU*	0.380	0.760	1.047	0.430	1.104	1.693	0.911	1.413	0.818	1.908	1.843	0.653	0.316	0.760	1.420	0.545
*NPC1*	0.859	0.938	0.918	0.930	1.197	1.055	0.492	1.268	0.976	0.964	0.984	0.993	0.609	1.055	1.527	1.235
*NPC2*	1.499	0.708	1.227	0.885	1.239	1.428	0.378	1.180	0.909	1.097	0.920	0.920	0.673	0.991	1.322	0.625
*OTC*	1.549	0.857	1.293	1.173	1.278	0.722	0.271	1.083	1.248	0.887	0.917	0.662	0.797	0.887	1.233	1.143
*PCBD1*	1.147	1.107	1.288	0.704	1.650	1.026	0.322	1.087	1.087	1.208	1.288	0.785	0.322	0.745	1.509	0.725
*PCCA*	1.408	0.730	1.285	1.209	1.194	0.602	0.368	0.908	1.294	0.896	1.040	0.925	0.737	0.843	1.462	1.097
*PCCB*	0.888	0.805	1.062	0.995	1.368	1.033	0.543	0.975	1.116	1.121	1.315	0.893	0.378	0.961	1.523	1.024
*PDSS1*	1.377	0.865	1.207	1.135	1.146	0.843	0.634	0.959	1.278	0.986	0.799	0.837	0.782	0.887	1.262	1.003
*PDSS2*	1.188	0.707	1.241	1.041	1.148	0.787	0.374	1.268	1.161	1.028	1.068	0.814	0.681	1.054	1.388	1.054
*PNP*	0.957	0.976	1.252	0.902	1.344	1.031	0.276	1.086	1.252	0.865	1.270	0.976	0.534	0.865	1.565	0.847
*PNPO*	0.897	0.856	1.223	0.530	0.897	1.406	0.591	1.386	1.366	1.141	1.488	0.815	0.346	0.876	1.508	0.673
*PTS*	1.721	0.732	1.062	1.465	0.915	0.696	0.439	0.805	1.281	0.805	0.989	0.952	1.025	0.622	1.574	0.915
*QDPR*	0.943	0.721	1.117	0.756	1.047	1.129	0.756	1.210	1.175	1.513	1.839	0.593	0.349	0.780	1.431	0.640
*SGSH*	0.467	1.151	0.956	0.342	1.173	2.004	1.178	1.329	1.000	1.587	1.267	0.707	0.276	0.942	1.160	0.462
*SLC2A1*	0.476	0.660	0.909	0.747	1.256	1.516	0.736	1.396	0.834	1.451	1.321	0.920	0.227	1.277	1.559	0.714
*SLC6A19*	0.395	0.950	0.832	0.697	1.311	1.613	0.840	1.479	0.874	1.286	1.361	0.857	0.294	1.395	1.345	0.471
*SPR*	0.795	0.795	0.938	0.285	0.815	1.427	1.039	1.610	0.978	1.875	1.631	0.754	0.224	0.795	1.631	0.408

### Relative Synonymous Codon Usage Analysis

RSCU, an index representing codon bias, is the ratio of the observed to the expected frequency of a codon coding for a particular amino acid among all synonymous codons (Deb et al., 2021). The RSCU values were obtained using informatics software developed by Puigbò et al. (2008) and available at http://genomes.urv.es/CAIcal/ (Puigbò et al., 2008). The length of the gene or amino acid composition does not affect RSCU values. Values above 1.6 and below 0.6 are considered overrepresented and underrepresented codons, respectively (Yu et al., 2021).

### The Parity Rule 2 Plot Analysis

Parity analysis shows the bias between AT and GC at the 3rd codon position. AT bias (A3%/[A3% + T3%]) and GC bias (G3%/[G3% + C3%]) are plotted on the ordinate and abscissa, respectively. Under ideal conditions, as per the rule of parity, in a strand of DNA, A = T and G = C provided there is no bias among mutation and selection (Sueoka, 1995). Therefore, at the center of the plot, where the value is 0.5, the selection and mutational forces are equal (Sueoka, 1999).

### Neutral Evolution Analysis

Neutrality plots are useful in quantifying mutational and other forces such as selectional forces (Khandia et al., 2019). Neutrality is derived by plotting %GC12 vs.%GC3. Here, the regression coefficient is considered the point of equilibrium between mutation and selection pressure (Sueoka, 1988). A slope value approximating 1 shows the dominance of mutational forces (Yu et al., 2021).

### Codon Adaptation Index

The CAI value expresses the adaptability and expression of any gene within the organism (Munjal et al., 2020). The values of CAI range between 0 and 1. Values approaching 1 indicate the gene has higher expressivity, while CAI values near zero represent lower expressivity (Sharp and Li, 1987). The calculations were performed using informatics software developed by Puigbò et al. (2008) and available at http://genomes.urv.es/CAIcal/. The codon usage table for *Homo sapiens* was obtained from the codon usage database^2^ encompassing 93487 coding sequences belonging to 40662582 codons.

### Intrinsic Codon Bias Index

The intrinsic codon bias index (ICDI) is an analogous index to the Nc value and is independent of optimal codons. The values of ICDI span between 0 and 1. A value of 1 indicates extremely high bias, while a value 0 indicates equal usage of codons. Values lower than 0.3 indicate comparatively low bias (Freire-Picos et al., 1994). The ICDI values were obtained using the formula provided by Freire-Picos et al. (1994).

### Nc Determination and Plotting Nc-GC3% Curve

The Nc-GC3 curve was plotted to evaluate the role of compositional constraints. Nc values, which reveal bias in codon usage, were determined using the CodonW 1.4.4 program. The lowest and the highest Nc values are 20 and 61, respectively (Munjal et al., 2020). An Nc value of 61 results from a situation when all the codons are used equally for coding amino acids, so no bias is detected. In contrast, a value of 20 results from only one codon being used among various synonymous codons, indicating the highest bias. In general, an Nc value less than 35 indicates a higher codon preference, and greater than 50 indicates random codon usage (Wang et al., 2018).

### Principal Component Analysis

In principal component analysis (PCA), a multivariate statistical approach in codon usage analysis, two major axes (axis 1 and axis 2) represent the two components contributing the most variation to the data. In the PCA, the RSCU values of each sequence were distributed into a 59-dimensional vector corresponding to the 59 synonymous codons. The stop, initiation (AUG), and tryptophan (UGG) codons were excluded.

### Protein Indices

Indices related to proteins were calculated using appropriate formulas available in the literature or software programs. The hydropathicity index GRAVY, with combined features of hydrophobicity or hydrophilicity, was calculated using the formula of Kyte and Doolittle (1982). Its value ranges between –2 and +2 for most proteins, with positive values indicating more hydrophobic proteins and vice versa. The AROMA value represents the distribution of aromatic amino acids (tryptophan, tyrosine, and phenylalanine) in the protein. Variation in these indices indicates the selection pressures. The PI or isoelectric point of any protein is the pH at which a protein has no net electrical charge. Hydrophobicity values were also determined as they have a role in determining protein-protein interactions (Young et al., 1994). This notion is further strengthened because few of the most extensive hydrophobic surfaces are found within the membrane proteins (Hagemans et al., 2015). The aliphatic index measures the relative volume occupied by aliphatic side chains (alanine, valine, isoleucine, and leucine) and was calculated by the formula given by Ikai (1980). The aliphatic index is positively correlated with the thermal stability of the protein. The % acidic, basic, and neutral amino acids were also calculated. All the indices were calculated using ExpasyProtparam tool (Gasteiger et al., 2005) or Peptide 2.0 tool available at https://www.peptide2.com/.

### Statistical Analysis

Correlation, regression, and correspondence analyses were performed and plotted in PAST4 software. Basic calculations, including sums and averages, were done in Microsoft Windows Excel.

## Results

### Compositional Analysis

**FIGURE 1:**
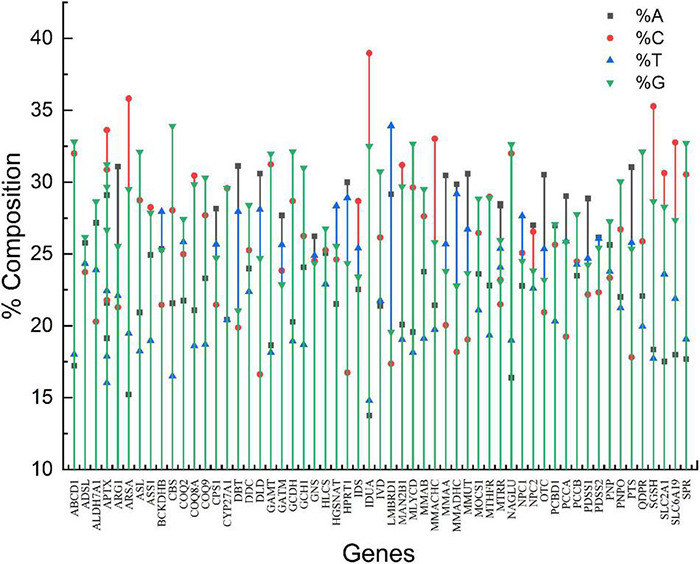
Stem graph for nucleotide composition of genes envisaged in present study.

The genes involved in neurodegeneration exhibited a widely variable compositional pattern. The % nucleotide composition of all four nucleotides is given in Figure 1. The %A ranged from 13.74% (*IDUA*) to 31.12% (*DBT*), %C ranged from 16.62 (*DLD*) to 38.98% (*IDUA*),%T ranged between 14.80% (*IDUA*) to 33.92%, and %G ranged between 19.57% (*LMBRD1*) and 33.91% (*CBS*). The nucleotide component C demonstrated the greatest range of variability (22.35%), while nucleotide G had the least (14.34%). Stem graph (Figure 1) showing the overall% of all the 4 nucleotides in different genes. Overall, %GC varied between 36.93% (*LMBRD1*) and 71.45% (*CBS*), while GC3 varied between 26.82% (*LMBRD1*) and 89.14% (*CBS*).

### GC Content Correlation With Protein Length

**FIGURE 2:**
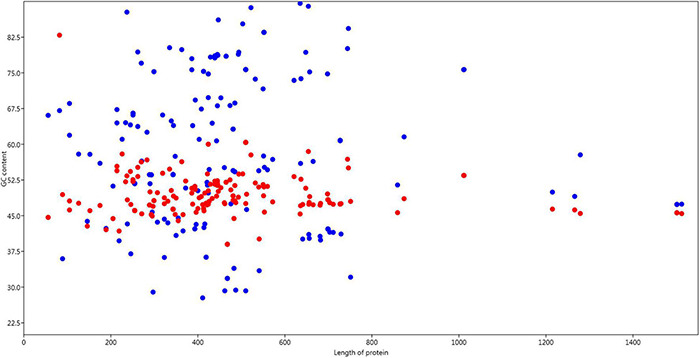
Relation of GC content GC12 and GC3 with the length of the protein. Red dots are GC12 composition and blue dots are GC3 position.

GC components (%GC12 and %GC3) were analyzed to determine their relationships with protein length. It was observed that, on average, %GC12 content was relatively constant and contributed to 40%-60% composition, while %GC3 content largely fluctuated towards both low and high values along the length of protein (Figure 2).

### Relationship of Compositional Properties and Codon Bias

A significant positive correlation was observed between Nc and different nucleotides. C1, A2, and G3 (*r* = –0.197, *p* < 0.05, *r* = –0.243 *p* < 0.01, *r* = –0.198 *p* < 0.01 respectively) were negatively correlated with Nc, while T, T1, T2, and C2 were positively correlated (*r* = 0.0.165, *p* < 0.05, *r* = 0.156 *p* < 0.05, *r* = 0.237, *p* < 0.01, *r* = 0.286 *p* < 0.001 respectively).

### Relationship Between Codon Usage Bias and Nucleotides at the Third Codon Position

**FIGURE 3:**
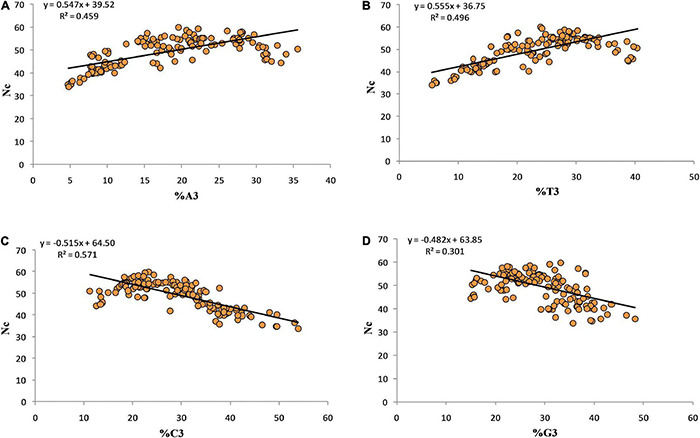
Regression analysis between the Nc and third position of codon.

Regression coefficients 0.547, 0.555, –0.515, and –0.482 were obtained for Nc-A3, Nc-T3, Nc-G3, and Nc-C3, respectively. The negative regression coefficient between Nc-G3 and Nc-C3 suggests a positive influence of C3 and G3 on CUB (Figure 3).

### Relationship Between Overall Composition and Composition at the Third Position of the Codon

**FIGURE 4:**
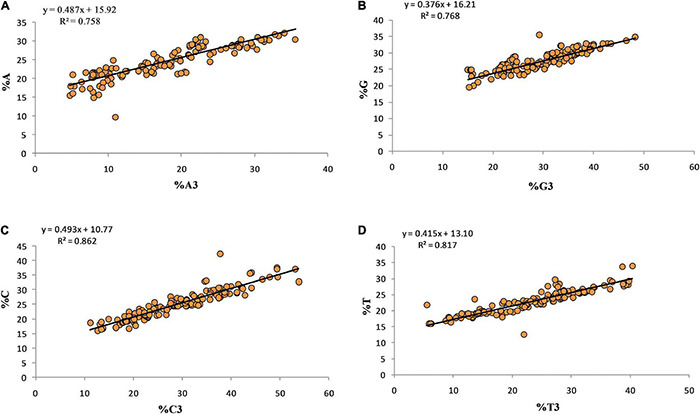
Regression analysis for overall nucleotide content and nucleotide content at third position.

Regression analysis between the overall nucleotide content (%A, %T, %C, %G) and their respective 3rd position of the codon shows the effects of compositional properties on mutational force (Figure 4). The nucleotides C and A contributed almost equally, 49.3 and 48.7%, while G contributed the least (37.6%) and toward mutational pressure.

### Effects of Dinucleotide Content

The frequency of dinucleotide occurrence is of great interest since some of the dinucleotide combinations significantly deviate from the expected value. The measure of this deviation is calculated as an odds ratio (frequency observed/expected). The CpG and TpA dinucleotide combinations are commonly underrepresented dinucleotides (Venter et al., 2001). Notably, we found that 23.33% of genes displayed unbiased TpA usage with an odds ratio of more than 0.78. In fact, in the IUDA gene, TpA was overrepresented (odds ratio 1.54). Nucleotide composition is also reported to play an important role in deciding the TpA or CpG content (Munjal et al., 2020); however, we report an exception to this as, despite lower GC content, the CpG dinucleotide exhibited its presence unbiased in *GCDH* and *MAN2B1* genes. Similarly, despite low AT content, the TpA dinucleotide was present in an unbiased manner *LMBRD1, MMAA, MOCS2* genes.

### Relative Synonymous Codon Usage Analysis

The RSCU value indicates the relative frequency of the codon. The codons with RSCU values above 1.6 and below 0.6 are overrepresented and underrepresented, respectively (Paul et al., 2018). Across the genes, GC ending codons had an RSCU value below 0.6 (TCG, CCG, ACG, and GCG codons are unrepresented with RSCU values below 0.6 in 81.66, 70, 68, and 72% of sequences, respectively). TA ending codons TTA, CTA, ATA, and GTA were also underrepresented with RSCU values below 0.6 in 71.67, 76.67, 71.67, and 68.33% of sequences, respectively. Among all codons, CTG and GTG had maximum RSCU values (average of 2.55 and 2.01, respectively) with the highest RSCU values of 4.73 and 3.27 for CTG and GTG, respectively. Table 3 lists the RSCU values of the genes analyzed in this study, highlighting the codons with the highest RSCUs for each amino acid.

**TABLE 3:** RSCU values of genes highlighting the codons with highest RSCU values.

			Name of genes																			
AMINOACIDS	One letter amino acid	Codon	*ABCD1*	*ADSL*	*ALDH7A1*	*APTX*	*ARG1*	*ARSA*	*ASL*	*ASS1*	*BCKDHA*	*BCKDHB*	*CBS*	*COQ2*	*COQ8A*	*COQ9*	*CPS1*	*CYP27A1*	*DBT*	*DDC*	*DLD*	GAMT
Phenylalanine	F	TTT	0.261	0.81875	1.2875	0.991304	1.010667	0.538667	0.667	0.533	0.556	1.485333	0.2708	1.385	0.4	1.111	1.258	0.667	1.238	1.254	1.54525	0.8445
	F	TTC	1.739	1.18125	0.7125	1.008696	0.989333	1.461333	1.333	1.467	1.444	0.514667	1.7292	0.615	1.6	0.889	0.742	1.333	0.762	0.746	0.45475	1.2
Leucine	L	TTA	0	0.639	0.456	0.229739	0.312	0.096333	0.07975	0.176	0	0	0	0.4325	0	0	0.54875	0.448	0.857	0.534571	1.54675	0
	L	TTG	0.255	1.07075	0.9115	1.024435	0.946667	0.386333	0.2165	0.353	0.545	1.779	0.3104	1.0265	0.774	0.514	1.291	0.537	1	0.518571	1.63125	0.316
	L	CTT	0.255	1.566	0.9865	1.421304	0.908667	0.29	0.40525	0.529	0.364	1.779	0.2438	0.9735	0.29	0.343	1.006	0.179	0.857	0.620429	1.45975	0
	L	CTC	1.66	0.55075	1.4425	0.922522	0.711	0.97	2.32675	2.118	1.091	0.753	1.0704	0.594	1.355	1.543	0.68575	1.433	1.143	0.522429	0.184	1.263
	L	CTA	0.191	0.23225	0.152	0.449522	1.304333	0.192667	0.2165	0	0.545	0.844	0.1224	0.918	0	0.686	0.63975	0.269	0.714	0.397429	0.49675	0
	L	CTG	3.638	1.9415	2.052	1.952304	1.817333	4.064333	2.755	2.824	3.455	0.844	4.2532	2.0555	3.581	2.914	1.8285	3.134	1.429	3.406714	0.6815	4.421
Isoleucine	I	ATT	0.375	1.4425	1.3545	1.628826	1.596667	0	1.01475	0.889	0.13	0.931	0.7616	1.5	0.857	0.75	1.74925	1.263	1.5	0.528857	1.7275	0.231
	I	ATC	2.531	1.2225	1.266	0.916043	0.862667	2.583333	1.816	2	2.739	1.034	2.2384	0.641	1.929	1.875	0.94925	1.579	0.5	1.987	0.58425	2.769
	I	ATA	0.094	0.335	0.379	0.455348	0.540667	0.416667	0.16925	0.111	0.13	1.034	0	0.859	0.214	0.375	0.3015	0.158	1	0.484571	0.68825	0
Valine	V	GTT	0.14	0.55275	0.9325	0.343913	0.366333	0.386667	0.284	0.133	0	1.69	0.2132	1.941	0.242	0.267	1.148	0.364	1.895	0.553429	2.03625	0
	V	GTC	0.772	0.7505	0.6425	0.521826	0.575333	1.213333	0.81325	1.467	1.524	0.818667	0.4034	0.4855	0.97	0	0.951	0.727	0.421	0.907571	0.38625	1.667
	V	GTA	0.14	0.6005	0.742	1.052826	0.522	0	0	0	0.381	0.673333	0.1062	0.3605	0	0.8	0.328	0.121	0.737	0.118	0.8505	0
	V	GTG	2.947	2.096	1.683	2.080826	2.536667	2.4	2.9035	2.4	2.095	0.818667	3.277	1.213	2.788	2.933	1.574	2.788	0.947	2.420857	0.727	2.333
Serine	S	TCT	0.638	1.774	1.156	1.711957	1.285667	1.266667	0.29625	0.5	1.5	1.8	0.486	1.1225	0.6	1.92	1.366	0.75	1.5	1.072	2.20575	0
	S	TCC	1.532	0.53375	1.0525	1.278174	0.885667	2.433333	0.85125	2.5	1.071	0.9	1.6088	1.762	2.55	1.2	1.366	1.75	0.938	1.494857	0.64425	2.4
	S	TCA	0.638	1.068	0.8425	0.635087	1.028667	0.216667	0.51075	0.25	0.857	0.9	1.1228	0.986	0.75	0.96	1.426	0.5	1.875	1.239286	2.10175	1.2
	S	TCG	1.66	0.16375	0	0	0	0	0.6815	0.25	0.429	0.3	0.536	0.252	0	0	0.119	0.75	0	0.181	0.2395	0.6
	S	AGT	0.128	1.29125	1.474	1.045217	1.085667	0.433333	1.022	0.25	1.071	1.2	0.3912	1.007	0.45	0.72	0.832	1.25	0.938	1.218429	0.40425	0
	S	AGC	1.404	1.169	1.474	1.329391	1.714333	1.65	2.638	2.25	1.071	0.9	1.8544	0.8705	1.65	1.2	0.891	1	0.75	0.793714	0.40425	1.8
Proline	P	CCT	0.457	1.01975	2.0955	1.797348	1.344	0.989333	0	1	0.5	2.013333	0.447	0.6855	0.914	0.857	1.123	1.143	1.857	1.372	1.163	0.211
	P	CCC	2.4	1.5815	0.381	0.857217	0.630667	1.744667	3.2365	1.4	2.833	0.56	1.8236	1.482	2.171	1.714	1.0105	1.6	0.571	1.184429	0.52975	2.316
	P	CCA	0.343	0.98025	1.1425	0.807478	2.025333	0.680667	0.382	1	0.5	1.32	0.647	1.1315	0.8	0.857	1.64225	0.8	1.429	1.098857	1.8545	0.842
	P	CCG	0.8	0.4185	0.381	0.537609	0	0.585333	0.382	0.6	0.167	0.106667	1.0824	0.701	0.114	0.571	0.22425	0.457	0.143	0.345143	0.453	0.632
Threonine	T	ACT	0.552	0.92225	1.726	1.175348	1.221333	0.829667	0.99075	0.667	0.286	2.340333	0.0944	1.34	0.571	0.632	1.187	0.8	1.655	0.856571	1.33625	0
	T	ACC	1.241	0.802	0.7845	0.989261	0.522333	2.249	1.853	2.889	1.714	0.237	1.9862	1.14	1.714	1.053	1.055	1.6	0.552	1.032857	0.46725	2.133
	T	ACA	0.828	2.11525	1.1755	1.835391	2.256333	0.626667	0.33025	0.222	0.857	1.422	0.3376	1.26	0.762	1.895	1.538	0.6	1.655	1.209429	1.86525	0.533
	T	ACG	1.379	0.1605	0.314	0	0	0.295	0.82575	0.222	1.143	0	1.5818	0.26	0.952	0.421	0.22	1	0.138	0.900571	0.331	1.333
Alanine	A	GCT	0.5	1.04975	1.0455	2.209	2.111	0.885	0.55475	0.571	0.539	0.867667	0.8096	0.9945	0.696	0.471	1.739	0.833	1.75	0.687	2.424	0
	A	GCC	2.6	1.4035	1.1365	0.899217	0.611	2.418333	2.26625	2.571	2.1575	1.319	1.8036	1.112	2.493	1.294	1.113	2.167	0.875	1.943857	0.4635	1.778
	A	GCA	0.25	1.27525	1.5	0.891783	1.277667	0.521	0.80875	0.571	0.5835	0.966333	0.4728	0.7615	0.464	1.294	1.043	0.5	1.25	0.927429	1.02325	0.444
	A	GCG	0.65	0.272	0.3185	0	0	0.175667	0.37025	0.286	0.719	0.846667	0.914	1.132	0.348	0.941	0.104	0.5	0.125	0.442	0.0895	1.778
Tyrosine	Y	TAT	0.37	1.44625	1.675	0.539174	0.611333	0.436	0.411	0.737	0.696	1.373667	0.25	0.714	0.571	0.667	0.842	0.632	2	0.293857	0.9045	0.25
	Y	TAC	1.63	0.55375	0.325	1.460826	1.388667	1.564	1.589	1.263	1.304	0.626333	1.75	1.286	1.429	1.333	1.158	1.368	0	1.706143	1.0955	1.75
Histidine	H	CAT	0.353	0.86975	0.6285	1.092609	0.722333	0.906333	0.55875	0.8	0.154	1.185	0.1144	0.9	0.476	1	1.034	0	1.4	0.716286	1.36075	0.25
	H	CAC	1.647	1.13025	1.3715	0.907391	1.277667	1.093667	1.44125	1.2	1.846	0.815	1.8856	1.1	1.524	1	0.966	2	0.6	1.283714	0.63925	1.75
Glutamine	Q	CAA	0.167	0.14575	0.251	0.586348	1.177667	0.113333	0.16875	0.588	0.138	0.244667	0.17	0.2665	0.125	0.182	0.53825	0.457	0.5	0.169571	0.7145	0
	Q	CAG	1.833	1.85425	1.749	1.413652	0.822333	1.886667	1.83125	1.412	1.862	1.755333	1.83	1.7335	1.875	1.818	1.46175	1.543	1.5	1.830429	1.2855	2
Asparagine	N	AAT	0.154	0.8065	1.024	0.852783	0.708	0.555667	0.7595	0.375	0.444	0.889	0.313	1.636	0.095	0.4	1.041	0.5	1.053	0.870714	1.43525	0.333
	N	AAC	1.846	1.1935	0.976	1.147217	1.292	1.444333	1.2405	1.625	1.556	1.111	1.687	0.364	1.905	1.6	0.959	1.5	0.947	1.129286	0.56475	1.667
Lysine	K	AAA	0.214	0.9	1.155	0.784174	0.678	0.25	0.597	0.667	0.222	1.361	0.248	0.908	0.303	0.222	0.947	0.25	1.22	1.041571	1.23525	0.6
	K	AAG	1.786	1.1	0.845	1.215826	1.322	1.75	1.403	1.333	1.778	0.639	1.752	1.092	1.697	1.778	1.053	1.75	0.78	0.958429	0.76475	1.4
Aspartic acid	D	GAT	0.5	1.01625	1.236	1.585957	0.853667	0.415333	0.471	0.75	0.87	1.537333	0.5724	0.824	0.6	0.933	1.253	0.737	1.417	0.633143	1.61275	0.545
	D	GAC	1.5	0.98375	0.764	0.414043	1.146333	1.584667	1.529	1.25	1.13	0.462667	1.4276	1.176	1.4	1.067	0.747	1.263	0.583	1.366857	0.38725	1.455
Glutamic acid	E	GAA	0.353	0.92925	1.0715	1.139652	1.562333	0.211	0.339	0.571	0.222	1.361333	0.2316	1.143	0.449	0.48	1.07175	0.562	1.786	0.828429	1.49625	0
	E	GAG	1.647	1.07075	0.9285	0.860348	0.437667	1.789	1.661	1.429	1.778	0.638667	1.7684	0.857	1.551	1.52	0.92825	1.438	0.214	1.171571	0.50375	2
Cysteine	C	TGT	0.444	0.61225	1.578	0.676826	2	0.420667	0.3	0.4	0.4	1.5	0.9272	1.653	0.444	0	1.2	0	1.429	0.654143	1.374	0
	C	TGC	1.556	1.38775	0.422	1.323174	0	1.579333	1.7	1.6	1.6	0.5	1.0728	0.347	1.556	2	0.8	2	0.571	1.345857	0.626	2
Arginine	R	CGT	0.61	1.2215	0	0.946391	0	1.048333	0.4155	0.6	0.171	0.190667	0.2234	0.9535	0.293	1.091	0.842	0.5	1.765	0.225429	0.26475	0.545
	R	CGC	2.034	1.40325	0.975	0.405478	0	1.457667	1.45375	1.8	1.714	0.688333	1.7364	1.3895	0.878	0.818	0.737	1.167	1.412	1.361714	0.76025	2.182
	R	CGA	0.102	0.9415	1.105	1.167087	0.979	1.166	0.5655	0.6	0.343	1.377333	0	0.676	0.439	0.818	0.632	0.667	1.059	0.427571	1.025	0
	R	CGG	2.237	1.40325	0.49	0.441391	0.489667	2.153667	2.527	1.5	2.571	1.106667	1.9084	1.5095	1.902	1.364	0.737	2.167	0	1.451	0	2.727
	R	AGA	0.102	0.893	1.84	1.537783	3.245	0	0	0	0.171	1.377333	0.1712	1.2315	0.439	0.545	1.684	0.667	1.412	1.625714	3.95	0
	R	AGG	0.915	0.1365	1.59	1.50187	1.287	0.174	1.0385	1.5	1.029	1.260333	1.9606	0.24	2.049	1.364	1.368	0.833	0.353	0.908571	0	0.545
Glycine	G	GGT	0.655	1.25225	0.764	0.204783	0.493667	0.447333	0.60175	0.516	0.516	0.566333	0.0912	1.0465	0.426	0.842	1.117	0.444	0.903	0.398571	1.61375	0.25
	G	GGC	2.036	0.8545	1.017	1.698696	0.889	1.827667	1.324	2.194	1.935	0.965667	1.5764	0.9505	2.043	1.474	0.937	1.556	0.774	0.884571	0.71675	2.5
	G	GGA	0.364	1.21675	1.7505	0.441348	1.926	0.494	0.4505	0.387	0.258	1.557	0.1824	1.429	0.426	0.632	1.261	0.667	1.677	1.344286	1.4405	0
	G	GGG	0.945	0.67625	0.4685	1.655261	0.691667	1.231	1.62375	0.903	1.29	0.910333	2.15	0.574	1.106	1.053	0.685	1.333	0.645	1.371857	0.22875	1.25
Phenylalanine	F	TTT	1.127	0.7635	0.91075	1.2	1.221125	1.2285	1.556	1.022	0.249667	1.005714	1.1215	0.545	0.737	0.667	0.8845	1.2	1.529	1.5	0.726	1.7145
	F	TTC	1.111	0.873	1.2365	1.08925	0.8	0.778875	0.7715	0.444	0.978	1.083667	0.994286	0.8785	1.455	1.263	1.333	1.1155	0.8	0.471	0.5	1.054333
Leucine	L	TTA	0.25	1.2665	0.0575	0.0715	0.677	0.4205	0.1895	0.571	0.206667	0.059333	0	1.4865	0.055	0	0.923	0	1.714	1.25	0.75	0.238
	L	TTG	0.5	1.1	0.462	0.87525	1.161	0.691125	0.91375	1.714	1.467333	0.178667	0.401429	1.215	0.44	0.448	0.231	1.167	0.429	0.75	0.844	0.559
	L	CTT	0.5	1.5535	0.404	1.435	0.29	1.09625	0.86075	1.143	1.044333	0.908333	0.992429	1.606	0.44	0.358	0.923	0.265	1.286	1.25	2.156	0.672556
	L	CTC	1	0.2865	1.385	1.127	1.161	1.56975	1.23175	0.857	0.988333	1.19	0.864857	0.4125	1.156	1.522	1.385	1.5395	0.643	1	0.656	1.502556
	L	CTA	0.25	0.5735	0	0.71775	0.484	0.149875	0.23875	0.286	0.282667	0.059333	0.242714	0.35725	0.22	0.179	0.231	0.7445	0.75	0.5	0.562	0.437333
	L	CTG	3.5	1.22	3.692	1.7735	2.226	2.0725	2.5655	1.429	2.010667	3.604667	3.498571	0.922	3.688	3.493	2.308	2.284	1.179	1.25	1.031	2.590333
Isoleucine	I	ATT	0.462	1.6075	0.8255	1.25475	1.05	1.225875	1.34875	2	0.554	0.211	0.88	1.69175	0.536	0.176	0.667	0.3165	1.286	1.75	1.558	0.688556
	I	ATC	2.538	1.24	2.1745	1.11825	1.5	1.27975	1.28575	0.4	1.714667	1.789	1.897571	0.3885	2.143	2.471	2	1.4165	1	0.5	0.577	1.794889
	I	ATA	0	0.1525	0	0.627	0.45	0.493375	0.3655	0.6	0.731333	0	0.222429	0.92	0.321	0.353	0.333	1.2665	0.714	0.75	0.865	0.516667
Valine	V	GTT	0.471	1.25	0.606	0.93775	1.032	0.83575	0.8835	0.8	1.222333	0.183333	0.424	1.61925	0.486	0.375	0	0.3895	1	1.565	1.447	0.449778
	V	GTC	1.412	0.5165	1.129	0.93775	1.032	0.986	0.84025	1.2	0.788	1.594333	1.245143	0.7935	0.649	0.625	1.091	0.3895	0.333	0.174	0.17	1.009667
	V	GTA	0	0.433	0.1745	0.914	0.387	0.200125	0.4695	0.8	0	0	0.469714	0.9135	0.378	0.125	0	0.932	1.167	1.043	1.021	0.293667
	V	GTG	2.118	1.8	2.091	1.21075	1.548	1.978125	1.80675	1.2	1.989667	2.222333	1.861714	0.6735	2.486	2.875	2.909	2.29	1.5	1.217	1.362	2.247111
Serine	S	TCT	0.462	1.1105	0.6255	0.28375	0.811	1.099125	2.33175	0	1.527	0.206333	1.404714	1.90275	0.2	0.353	0.632	0.4145	2.069	1.304	2.049	1.096778
	S	TCC	1.846	2.513	1.163	0.76575	1.622	1.558	1.1205	1.091	1.466667	2.889	1.385857	0.362	1.3	1.235	0.632	1.243	0.207	0.261	1.024	1.623556
	S	TCA	0.923	1.4025	0.3585	0.47125	0.973	0.83825	0.15575	1.091	0.743	0	0.455286	1.179	1	0.882	0.316	1.657	1.862	1.565	1.61	0.594
	S	TCG	0.462	0	1.7915	1.14875	0.162	0.099	0.543	0	0.447667	0.77	0.211857	0.198	0.9	0.882	1.895	0	0.207	0.522	0	0.174556
	S	AGT	0.462	0.351	0.537	0.383	1.622	1.099125	0.955	2.182	0.606333	0	1.312286	1.377	0.5	0.353	0.947	0.4145	1.241	1.826	0.732	0.865778
	S	AGC	1.846	0.6235	1.524	2.948	0.811	1.306375	0.8945	1.636	1.209667	2.134667	1.23	0.981	2.1	2.294	1.579	2.2715	0.414	0.522	0.585	1.645667
Proline	P	CCT	0.522	1.287	1	0.8095	0.973	1.209625	1.296	1.778	1.538333	0.512	1.611429	1.575	0.824	0.857	1.143	1.149	1.053	1.75	1.778	0.843444
	P	CCC	2.261	1.192	2.25	1.714	0.865	1.679	0.898	0	1.280333	1.580667	1.290571	1.31225	1.765	1.286	1.429	1.5845	0.632	0.5	0.556	1.626
	P	CCA	0.87	1.4515	0.5	0.33325	2.054	0.5995	1.55975	2.222	0.687333	0.563667	0.410286	1.11275	0.353	0.286	1.429	1.0465	2.105	1.75	1.556	0.774667
	P	CCG	0.348	0.069	0.25	1.143	0.108	0.512	0.246	0	0.493333	1.343	0.688143	0	1.059	1.571	0	0.2205	0.211	0	0.111	0.755889
Threonine	T	ACT	0.533	1.094	0.1965	0.5455	1.714	1.047875	1.297	2.545	0.623333	0.22	1.165	1.896	0.491	0.6	0.235	1.2955	1.913	2.588	1.622	0.938111
	T	ACC	2.4	0.983	2.158	1.212	1.029	1.455	1.31375	0.727	2.256333	1.598	1.578857	0.606	1.684	1.4	1.176	0.6135	0.87	0.235	0.973	1.716778
	T	ACA	0.533	1.4705	1.072	0.879	1.029	1.06225	0.714	0.727	0.561	0.886667	1.204286	1.21125	1.263	1	1.647	1.7275	1.217	1.176	1.405	1.270778
	T	ACG	0.533	0.453	0.574	1.3635	0.229	0.43575	0.6755	0	0.56	1.295	0.052	0.2865	0.561	1	0.941	0.3635	0	0	0	0.074111
Alanine	A	GCT	0.552	2.0785	0.6275	0.5855	1.29	1.310875	1.30925	1.2	0.949667	0.509	1.085429	1.962	0.524	0.308	0.727	1.3775	1.333	1.524	2.087	1.194778
	A	GCC	1.379	0.785	2.631	1.3665	1.161	1.588875	1.5505	1.2	2.250667	1.575667	1.865571	0.54225	2.095	1.846	2.364	2.1245	1.037	0.762	0.58	1.945
	A	GCA	0.552	0.896	0.229	0.876	1.161	0.6725	0.7455	1.2	0.69	0.283333	0.510714	1.28975	0.524	0.205	0.182	0.498	1.333	1.714	1.159	0.503333
	A	GCG	1.517	0.24	0.513	1.1715	0.387	0.42775	0.3945	0.4	0.109667	1.632	0.538	0.206	0.857	1.641	0.727	0	0.296	0	0.174	0.357
Tyrosine	Y	TAT	0.571	0.6125	0.769	1.05	1.154	0.995	1.067	1.455	1.262667	0.031667	0.661	1.20125	0.529	0.462	1.143	0	1.143	1.429	1.263	1.186667
	Y	TAC	1.429	1.3875	1.231	0.95	0.846	1.005	0.933	0.545	0.737333	1.301667	1.339	0.79875	1.471	1.538	0.857	2	0.857	0.571	0.737	0.813333
Histidine	H	CAT	0.6	1.139	0.325	1.275	1.091	0.757625	0.64825	1.6	1.199	0.498667	0.563857	1.143	0.296	0.769	1.333	0.8035	0.769	1.75	1.067	0.974444
	H	CAC	1.4	0.861	1.675	0.725	0.909	1.242375	1.35175	0.4	0.801	1.501333	1.436143	0.857	1.704	1.231	0.667	1.1965	1.231	0.25	0.933	1.025556
Glutamine	Q	CAA	0	0.3095	0.105	0.8445	0.5	0.44475	0.127	0	0.578	0.108333	0.227429	1.64375	0.248	0.25	0.364	0.5035	1	0.889	0.588	0.175222
	Q	CAG	2	1.6905	1.895	1.1555	1.5	1.55525	1.873	2	1.422	1.891667	1.772571	0.35625	1.752	1.75	1.636	1.4965	1	1.111	1.412	1.824778
Asparagine	N	AAT	0.667	1.021	0.6655	0.36325	0.971	0.52075	0.97025	2	0.709667	0.217667	1.227714	1.524	0.708	0.267	1	1.1	2	1.333	1.44	0.477778
	N	AAC	1.333	0.979	1.3345	1.63675	1.029	1.47925	1.02975	0	1.290333	1.115667	0.772286	0.476	1.292	1.733	1	0.9	0	0.667	0.56	1.522222
Lysine	K	AAA	0.6	1.1665	0.4375	0.47475	0.667	0.6405	0.935	1	0.655333	0	0.280714	1.26575	0.583	0.545	0.8	0.343	1.533	1.286	1.143	0.562556
	K	AAG	1.4	0.8335	1.5625	1.52525	1.333	1.3595	1.065	1	1.344667	1.333333	1.719286	0.73425	1.417	1.455	1.2	1.657	0.467	0.714	0.857	1.437444
Aspartic acid	D	GAT	0.545	1.027	0.364	1.4275	0.621	0.679875	0.78525	1.143	1.142667	0.132	0.748	1.62325	0.731	0.364	0.545	0.641	1.053	1.467	1.55	0.971111
	D	GAC	1.455	0.973	1.636	0.5725	1.379	1.320125	1.21475	0.857	0.857333	1.868	1.252	0.37675	1.269	1.636	1.455	1.359	0.947	0.533	0.45	1.028889
Glutamic acid	E	GAA	0.471	1.003	0.286	0.4805	0.9	0.934	0.98325	1.556	1.067667	0.395	0.374571	1.55825	0.311	0.579	0.824	0.5225	1.083	1.667	1.709	0.528556
	E	GAG	1.529	0.997	1.714	1.5195	1.1	1.066	1.01675	0.444	0.932333	1.605	1.625429	0.44175	1.689	1.421	1.176	1.4775	0.917	0.333	0.291	1.471444
Cysteine	C	TGT	0	0.6445	0.7335	1.08325	0.857	1.2455	0.96725	1.5	0.623667	0.911	1.047286	1.69725	0.5	1.143	0	1.1	2	1.111	2	0.650889
	C	TGC	2	1.3555	1.2665	0.91675	1.143	0.7545	1.03275	0.5	1.376333	1.089	0.952714	0.30275	1.5	0.857	2	0.9	0	0.889	0	1.349111
Arginine	R	CGT	0	1.2145	0.185	0	0.414	0.191125	0.186	0.5	0.335667	0.26	0.479143	0.6785	0.429	0.167	1.333	0.9	0.462	0.462	1.143	0.381
	R	CGC	2.667	0.6855	1.2925	0.675	0.621	0.411375	1.388	0.5	1.672	3.338667	1.226286	0.30525	2.486	2.333	1	0.9	0.231	0.462	0.429	0.550667
	R	CGA	0.667	1.4145	0.3695	0.15	0.621	0.321375	1.3385	1	0.647667	0.568333	0.813	0.91475	0.429	0.333	0	1.8	1.385	1.846	1.286	0.695667
	R	CGG	2.667	0.643	2.031	3	1.448	1.186125	0.5765	0	0.933333	0.891667	1.818286	0.4415	1.543	2	1	0.9	0.462	0	0.286	2.285556
	R	AGA	0	1.0715	1.0145	0.675	1.241	2.281375	1.00575	1.5	0.738667	0.173	0.206286	2.44	0.429	0.5	1.333	1.2	1.846	2.308	1.714	1.167444
	R	AGG	0	0.9715	1.108	1.5	1.655	1.608625	1.5055	2.5	1.672	0.768333	1.457	1.22	0.686	0.667	1.333	0.3	1.615	0.923	1.143	0.919556
Glycine	G	GGT	0.444	0.65	0.6255	0.51325	0.718	0.372875	0.49975	0.5	0.617667	0.737333	0.535857	1.24575	0.629	0.488	0	0.3095	0.914	1.474	1.103	0.621556
	G	GGC	1.481	1.333	1.6405	1.84175	1.026	1.71225	1.08075	1	1.548333	2.357	1.913143	0.88575	1.6	1.756	1.565	1.6905	0.343	0.211	0.483	1.433
	G	GGA	0.593	1.85	0.4325	0.526	1.128	1.095125	1.555	2	1.054667	0.276	0.379714	1.55725	0.8	0.39	1.043	0.143	2.057	1.684	2	0.926556
	G	GGG	1.481	0.1665	1.301	1.119	1.128	0.819625	0.86475	0.5	0.779333	0.629667	1.171	0.3115	0.971	1.366	1.391	1.857	0.686	0.632	0.414	1.018889
Phenylalanine	F	TTT	0.71	1.344667	1.2578	0.529	1.011	0.355333	1.467	0.833333	1.267	1.259	1.241	1.833	1.067	0.714	1.5	0.9285	0.656667	0.421	0.14	0
	F	TTC	1.29	0.655333	0.7422	1.471	0.989	1.644667	0.533	1.166667	0.733	0.741	0.759	0.167	0.933	1.286	0.5	1.0715	1.343333	1.579	1.86	2
Leucine	L	TTA	0.091	0.901333	0.7112	0	0.403	0	0.612	0	1.130273	0.1605	0.836333	0.706	0.692	0	0	0.286	0.128	0	0.08	0.125
	L	TTG	0.455	0.869333	0.5958	0.552	1.03	0	1.469	0.166667	1.081273	1.282	0.889	1.176	0.231	0.522	1.091	0.571	0.256667	0	0.08	0.5
	L	CTT	0.727	0.911333	1.5368	0.414	0.94	0.308	0.612	0.933333	0.966273	0.795	1.673	1.176	0.923	1.043	1.636	0.286	0.111	0.305	0	0.5
	L	CTC	1.227	1.101	1.1924	0.897	0.896	1.677	0.857	0	0.657636	1.199	0.882333	0.706	1.154	0.783	1.091	2	2.073333	1.322	1.92	0.75
	L	CTA	0.1355	0.326667	0.625	0.276	0.537	0.661667	0.612	0.166667	0.827182	0.481	0.534	0.706	0.462	0.261	0.545	0.571	0.256667	0.305	0.16	0
	L	CTG	3.364	1.890333	1.3388	3.862	2.194	3.354	1.837	4.733333	1.337273	2.0835	1.185	1.529	2.538	3.391	1.636	2.286	3.174667	4.068	3.76	4.125
Isoleucine	I	ATT	0.1715	1.460667	1.2336	0	1.2	1.166667	0.938	0	1.772182	0.823	1.403333	1.143	0.5	0.75	1.8	0.548	0.424333	0.324	0.367	0
	I	ATC	2.615	0.981333	0.675	2.647	1.2	1.222	1.688	2.416667	0.841273	1.933	0.556333	1.429	2.25	0.75	1.2	2.0715	2.575667	2.514	2.633	3
	I	ATA	0.213	0.558	1.0914	0.353	0.6	0.611333	0.375	0.583333	0.386545	0.2435	1.040333	0.429	0.25	1.5	0	0.381	0	0.162	0	0
Valine	V	GTT	0.211	1.131	1.4334	0.308	0.571	0.705667	0.8	0	1.209818	1.2675	1.629667	0.333	1.043	0.286	1.333	1.658	0.147667	0.455	0.075	0.211
	V	GTC	1.05	1.010667	0.2592	1	1.295	0.78	0.533	0.755667	0.755	1.3065	0.398	0.5	0.87	1.429	0	0	1.554	0.818	1.208	0.632
	V	GTA	0.211	0.384	0.9694	0.077	0.305	0.231333	1.067	0.489	0.594182	0.2375	0.953667	1.167	0.348	0	1.111	0.265	0.103333	0	0.075	0
	V	GTG	2.528	1.474	1.3378	2.615	1.829	2.282667	1.6	2.755667	1.441	1.188	1.018667	2	1.739	2.286	1.556	2.077	2.195333	2.727	2.642	3.158
Serine	S	TCT	0.431	1.186	1.4054	0.625	1.03	1.847333	1.364	0	1.543455	1.612	1.750333	0.818	1.385	1.125	0	0.716	0.281333	0.514	0.462	0.9
	S	TCC	2.105	1.489333	0.8674	2.25	1.273	0	1.091	1.9	0.425545	0.7165	0.584	1.364	0.462	1.875	1.5	1.0735	1.285	2.4	3.231	2.4
	S	TCA	0.373	1.054667	1.6492	0.125	0.727	0.111	1.364	1.233333	1.541818	0.984	1.004667	1.091	0.462	0	0	0.716	0.361333	0.686	0.231	0.3
	S	TCG	0.3645	0.065333	0.3296	0.125	0.545	0	0	0	0.406091	0	0.386333	0.545	0	0	0	0.516	0.501667	0.343	0.346	0.9
	S	AGT	1.177	1.329	0.7946	0.625	1.273	0.716667	0.818	0	0.981545	0.8955	1.536333	0.955	2.308	1.875	1.5	0	0.300667	0.686	0.115	0.6
	S	AGC	1.55	0.875667	0.953	2.25	1.152	3.325	1.364	2.866667	1.101364	1.7915	0.738	1.227	1.385	1.125	3	2.979	3.270333	1.371	1.615	0.9
Proline	P	CCT	0.674	1.560667	1.2456	0.636	1.538	0.926667	1.714	2.533333	1.722909	2.04	0.655	1.333	1.333	1.684	1.6	0.889	0.325667	0.522	0.625	1.333
	P	CCC	2.026	1.147	0.7308	1.636	0.923	1.544667	0.286	0	0.663	0.9415	1.073333	1	1.333	1.474	0.8	0.889	2.092667	2.783	2	1.333
	P	CCA	0.939	0.853	1.5456	0.818	1.169	1.22	1.714	1.466667	0.926091	0.7845	1.728	0.667	1.333	0.842	1.6	0.444	0.414667	0.522	0.25	0.667
	P	CCG	0.3605	0.439333	0.478	0.909	0.369	0.309	0.286	0	0.688	0.234	0.544	1	0	0	0	1.778	1.166667	0.174	1.125	0.667
Threonine	T	ACT	0.8	1.154333	0.8684	1.103	1	0.333333	0.833	0.666667	1.221182	0.741	0.753667	1.75	1.867	0.727	1.333	0.8705	0.887	0.615	0	1
	T	ACC	2.267	1.114667	1.3526	1.931	1.562	3.055333	1	0.666667	1.077727	1.778	1.145	1.25	1.333	1.818	0	1.757	2.137667	1.846	1.943	2
	T	ACA	0.533	1.469333	1.4948	0.276	0.938	0.611333	1.667	2	1.420909	1.037	2.023	0.5	0.8	0.364	0.667	1.1215	0.501	0.769	0.914	0.5
	T	ACG	0.4	0.262	0.2842	0.69	0.5	0	0.5	0.666667	0.280273	0.444	0.078333	0.5	0	1.091	2	0.251	0.475	0.769	1.143	0.5
Alanine	A	GCT	1.122	1.267667	0.8508	0.589	0.864	1.095333	1.538	1.344333	1.304727	0.794	1.007667	1.818	0.909	2	1.714	1.676	0.581333	0.941	0.25	0.462
	A	GCC	1.606	1.432333	1.413	2.021	1.818	1.642667	0.462	1.998667	0.769455	1.3555	1.487333	0.97	1.273	1.25	0	1.0285	2.305333	2.235	3	2.462
	A	GCA	0.7825	1.185667	1.613	0.589	0.773	0.714	1.692	0.657	1.574455	1.3215	1.076	1.091	1.818	0.5	1.143	0.648	0.314	0.471	0.25	0.615
	A	GCG	0.489	0.114	0.1232	0.8	0.545	0.547333	0.308	0	0.351455	0.529	0.429	0.121	0	0.25	1.143	0.648	0.799333	0.353	0.5	0.462
Tyrosine	Y	TAT	0.2795	0.92	1.58	0.667	0.815	1	1.273	0	1.278636	0.923	0.750333	1.333	1.111	0.667	1.25	0.667	0	0.923	0.5	1
	Y	TAC	1.7205	1.08	0.42	1.333	1.185	1	0.727	2	0.721364	1.077	1.249667	0.667	0.889	1.333	0.75	1.333	2	1.077	1.5	1
Histidine	H	CAT	0.3305	1.080333	1.3742	0.286	0.632	2	0.889	1.066667	1.476182	0.727	0.962333	1.385	0.75	0.667	1.2	1.5	0.188	0.8	0.333	0
	H	CAC	1.6695	0.919667	0.6258	1.714	1.368	0	1.111	0.933333	0.523818	1.273	1.037667	0.615	1.25	1.333	0.8	0.5	1.812	1.2	1.667	2
Glutamine	Q	CAA	0.108	0.647667	1.0572	0.229	0.327	0.655	0.824	0.785667	0.881091	0.812	0.457667	0.632	0.615	0.545	0.5	0	0.338333	0.286	0.091	0.364
	Q	CAG	1.892	1.352333	0.9428	1.771	1.673	1.345	1.176	1.214333	1.118909	1.188	1.542333	1.368	1.385	1.455	1.5	2	1.661667	1.714	1.909	1.636
Asparagine	N	AAT	0.5905	1.195667	1.0872	0.696	1.049	1.454	1.375	0.244333	1.124727	1.238	1.556333	1.158	0.545	1	1.455	0.452	0.492333	0.429	0.148	0.2
	N	AAC	1.4095	0.804333	0.9128	1.304	0.951	0.546	0.625	1.755667	0.875273	0.762	0.443667	0.842	1.455	1	0.545	1.548	1.507667	1.571	1.852	1.8
Lysine	K	AAA	0.3185	0.799667	0.7808	0.5	1.286	1.103333	1.154	1.133333	1.175	0.5	0.98	0.762	0.833	1.143	1.818	0.5165	0.444333	0.375	0.125	0.714
	K	AAG	1.6815	1.200333	1.2192	1.5	0.714	0.896667	0.846	0.866667	0.825	1.5	1.02	1.238	1.167	0.857	0.182	1.4835	1.555667	1.625	1.875	1.286
Aspartic acid	D	GAT	0.3695	1.000667	1.2478	0.37	0.783	1	1.412	0.4	1.392818	1.062	1.167	0.75	1.077	0.833	1.333	0.9445	0.252667	0.571	0	0.4
	D	GAC	1.6305	0.999333	0.7522	1.63	1.217	1	0.588	1.6	0.607182	0.938	0.833	1.25	0.923	1.167	0.667	1.0555	1.747333	1.429	2	1.6
Glutamic acid	E	GAA	0.476	1.142667	1.0536	0.2	0.958	1.314667	1.2	0.918667	1.333091	0.6935	1.408667	0.857	1.333	0.417	1.333	1.1	0.232	0.333	0.148	0.308
	E	GAG	1.524	0.857333	0.9464	1.8	1.042	0.685333	0.8	1.081333	0.666909	1.3065	0.591333	1.143	0.667	1.583	0.667	0.9	1.768	1.667	1.852	1.692
Cysteine	C	TGT	0.5225	0.843	1.1	0.5	1.184	0.355333	1.333	2	1.417273	1.091	1.394333	1	2	1	0	0.5	0.481333	0.667	0.125	0.8
	C	TGC	1.4775	1.157	0.9	1.5	0.816	1.644667	0.667	0	0.582727	0.909	0.605667	1	0	1	2	1.5	1.518667	1.333	1.875	1.2
Arginine	R	CGT	0.6015	0.235667	0.2304	0.245	0.391	4.4	0.8	0.571333	0.594182	1.235	0.594	1.579	1.412	0.571	1.2	0	0.785667	0.571	0	0.75
	R	CGC	1.504	0.539667	0.68	2.082	1.174	0	0	0	0.457182	0.882	0.586667	0.632	0	2.286	3.6	1.333	2.241333	2.571	1.667	1.875
	R	CGA	0.823	0.740667	0.8304	0.857	1.043	0	1.6	0	0.891364	0.882	1.702667	0.316	1.412	0.857	1.2	1.333	0.241333	0.286	0.667	0.75
	R	CGG	1.504	1.169	0.6096	1.714	1.304	0	0.8	1.285667	0.583182	0.882	0.954	0.947	0.706	1.429	0	1.333	1.821333	2.571	2	1.875
	R	AGA	0.7515	1.737667	2.28	0.122	0.913	0.8	2	1.571333	2.795545	1.059	2.016333	1.895	0.353	0.857	0	0	0.503	0	0.333	0.375
	R	AGG	0.816	1.576667	1.3696	0.98	1.174	0.8	0.8	2.571667	0.678636	1.059	0.146667	0.632	2.118	0	0	2	0.408	0	1.333	0.375
Glycine	G	GGT	0.689	0.523	0.5788	0.133	0.346	1.162	1.091	0.222333	0.919818	0.758	1.110333	0.8	1	0.571	0.8	0.64	0.541667	0.696	0.174	0.174
	G	GGC	1.9365	1.174333	0.7832	2.333	1.481	1.028667	0.727	2.577667	1.094182	1.052	0.522667	0.64	1.143	1.333	0.8	1.44	1.701	2.348	2.348	2.261
	G	GGA	0.6445	1.492667	1.8558	0.4	1.383	1.676	1.455	0	1.125364	1.2635	1.498667	1.6	1.143	0.762	1.6	0.96	0.752667	0.348	0.435	0.348
	G	GGG	0.7305	0.81	0.7832	1.133	0.79	0.133333	0.727	1.2	0.860727	0.9265	0.868333	0.96	0.714	1.333	0.8	0.96	1.004667	0.609	1.043	1.217

### Analysis of Rare Codons

**FIGURE 5:**
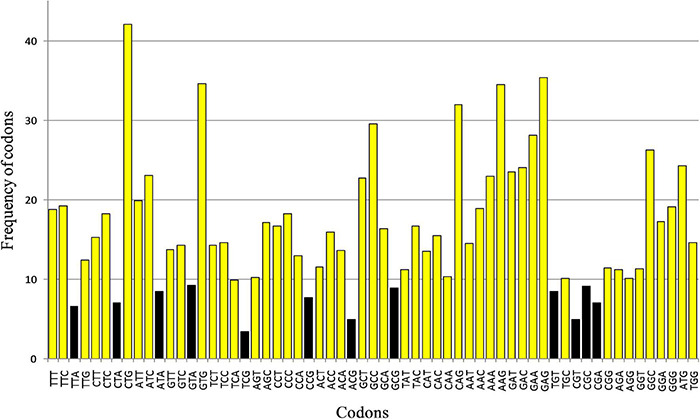
Rare codons for the neurodegeneration associated gene transcripts. The “rare codon” was defined by calculating the frequency of occurrence of all codons in coding sequences (threshold selected <1% viz. less than 10 in 1,000).

The codons with a frequency of occurrence below 1% were considered rare codons. Codons TTA, CTA, ATA, GTA, TCG, CCG, ACG, GCG, CGT, CGC, CGA, and TGT were rare codons (Figure 5). The codon usage is tissue-specific and in the brain, the codon usage might be different from other tissues. We adapted per million frequencies of codon bias and per million frequencies of the codon counts multiplied by the respective expression; the value would be called codonome bias hereafter in the human brain dataset from the works of Piovesan et al. (2013). A very high statistically significant positive correlation (*r* = 0.665, *p* < 0.001) between codon bias for genes envisaged and codonome bias for brain-specific genes was observed, revealing the presence of tissue and cell-specific selective pressure on gene-related to neurodegeneration with metabolic consequences in present study. Changes in the expression profile of isoacceptor tRNAs might result from adaptation and selection to changes in transcriptome codon usage (Dittmar et al., 2006). Also, tRNA-Arg-TCT enrichment is present and it is suggestive of a tissue-specific role of tRNA in translation (Torres et al., 2019).

### The Parity Rule 2 Plot Analysis

**FIGURE 6:**
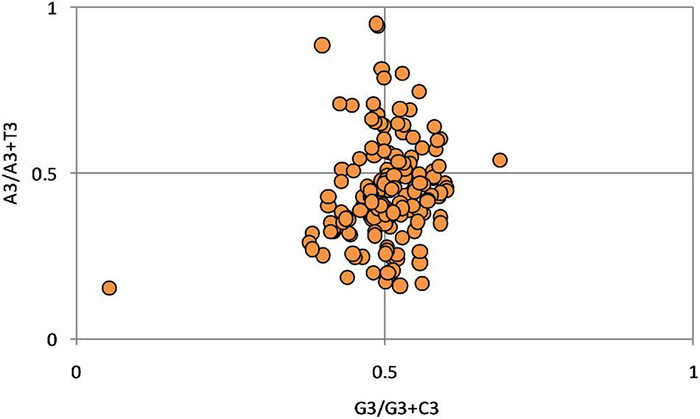
Parity plot generated using A3/(A3 + U3) as abscissa and G3/(G3 + C3) as the ordinate. The plot exhibit the preference of T over A and G over C.

As per the parity rule, in the absence of mutational force or selection force on a gene’s codon usage, the base content follows Chargaff’s rule: A = T and G = C. The A3%, T3%, C3%, and G3% (nucleotide content at the third position of the codon) were calculated to determine the A3/(A3 + U3) serving as AT bias and G3/(G3 + C3) serving as GC bias. When A3/(A3 + U3) is plotted against G3/(G3 + C3) on the abscissa and the ordinate, respectively, if the values are 0.5, then all the data will be located in the center (Young et al., 1994). However, the codon usage is generally governed by either of these or both the selection and mutation pressure and other forces such as compositional pressure. In the present study, the results (Figure 6) show that the average position of x = 0.449 ± 0.152 (AT bias) and y = 0.511 ± 0.054 (GC bias). Hence T is preferred over A, and G is preferred over C.

### Degree of Codon Bias

The Nc value determines the degree of bias. The higher the Nc, the lower the bias. A gene with an Nc value less than 35 generally has a strong codon bias (Sheikh et al., 2020), while the gene with an Nc value of greater than 50 has a random choice of codon, indicating the least codon bias. Nc values between 35 and 50 demonstrate moderate bias (Wang et al., 2018). In the present study, the Nc value ranged between 33.9 and 59.9, indicating that the genes exhibited a wider range of codon bias. An Nc < 35 was observed for 2.17% of transcripts, representing a very high bias. Furthermore, 40.21% had moderate bias (Nc 35-50), and 56.60% of transcripts displayed low bias (Nc > 50).

### The Effect of Compositional Constraint in Shaping Codon Usage

**FIGURE 7:**
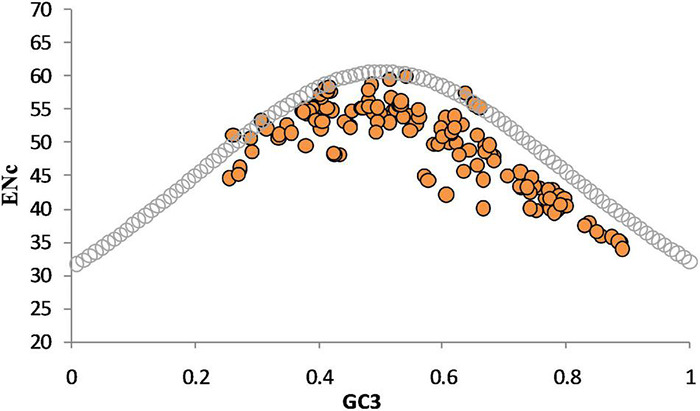
The relationship between compositional constraint and codon usage bias. ENc-GC3 curve indicates the presence of selection and mutational forces on codon bias of genes. Data points far from the standard curve indicate action of forces other than compositional constraints acting on codon usage choices.

The Nc-GC3 curve was plotted to elucidate further the effects of mutational, selectional, or compositional constraints on codon usage. If the codon usage was solely driven by %GC content present at the third position of the codon, then all the data points will lie on the GC3 curve. If a gene is subjected to translational selection, the data points will lie well below the expected curve (Sablok et al., 2011). In the present case, all low Nc points were well below the curve, suggesting forces other than compositional constraints like selectional forces affected codon usage (Figure 7).

### Neutrality Analysis for Quantitation of Mutation and Selection Pressure

**FIGURE 8:**
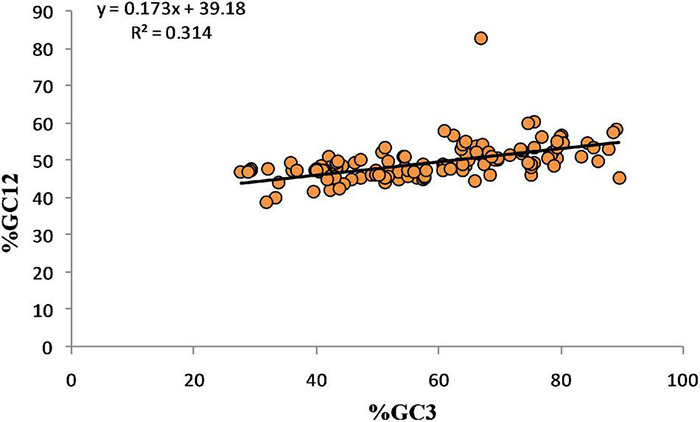
Neutrality plot analysis revealed 17.25% mutational forces and 82.68% selection forces acting on 60 genes related to neurodegenerative disorders.

Although the Nc-GC3 plot can demonstrate the key factors responsible for shaping codon bias, it cannot quantitate the directional pressure and selection pressure. For the same neutrality plot is required, constructed using the %GC3 and %GC12 content of genes. The GC3 content of the genes varied from 27.7 to 89.6%, while GC1 and GC2 varied from 41.7–89% to 30.1–76.8%, respectively. The linear regression model of GC12% on GC3% indicated (GC12%) = 0.1732 (GC3%) + 39.18 with R^2^ = 0.315. It suggests that a 31.5% variance in GC12 is introduced by GC3. The regression coefficient is 17.25%, indicating that mutational forces contribute 17.32%, while selection and other factors contribute 82.68%. The results indicate that selection pressures are dominant over mutational forces (Figure 8).

### Effect of Mutational Forces on Gene Expression

The CAI is an index for gene expressivity. The higher the CAI value, the greater the expressivity of the gene (Kumar et al., 2021). CAI and %A3, %T3, %C3, and %G3 were analyzed by regression analysis to determine the effect of mutational forces on gene expression. The results indicate that for all four nucleotides, an almost straight line is obtained as a regression curve (regression coefficients of –0.0047, –0.0045, 0.004, and 0.004 for A3, T3, C3, and G3, respectively). These results indicate that substantially fewer mutational forces were at work, and selectional forces majorly influence gene expression.

### Intrinsic Codon Bias Index

An ICDI estimates codon bias where optimal codons are unknown (Rodríguez-Belmonte et al., 1996). The average ICDI value was 0.182, and vales were well correlated with Nc values. According to Freire-Picos et al. (1994), if the ICDI value is greater than 0.5, there is a high level of bias. We found generally low levels of bias. The lowest ICDI value was 0.05 for 5-methyltet*r*ahydrofolate-homocysteine methyltransferase (*MTR*) transcript variant 2, while the highest was 0.501 for solute carrier family 6 member 19 (*SLC6A19*). However, the average ICDI was 0.182 ± 0.008, indicating a generally low bias.

### The Interrelationship Between Codon Usage Bias and Properties of Protein

Protein indices including protein length, GRAVY, AROMO, PI, hydrophobicity index, aliphatic index, instability index, and percent acidic, basic, and neutral amino acids were estimated and subjected to correlation analysis (Barbhuiya et al., 2020). The correlation analysis between the codon usage bias and various protein indices revealed that Nc was positively associated with PI (*r* = 0.176; *p* < 0.05), acidic (*r* = 0.216; *p* < 0.01), and basic (*r* = 0.347; *p* < 0.0001) protein residues. The Nc was negatively correlated with GRAVY (*r* = –0.173; *p* < 0.05), hydropathicity (*r* = –0.220; *p* < 0.01) and neutral amino acids (*r* = 0.146; *p* < 0.05). Nc and protein length did not correlate to codon usage bias.

### Principal Component Analysis

**FIGURE 9:**
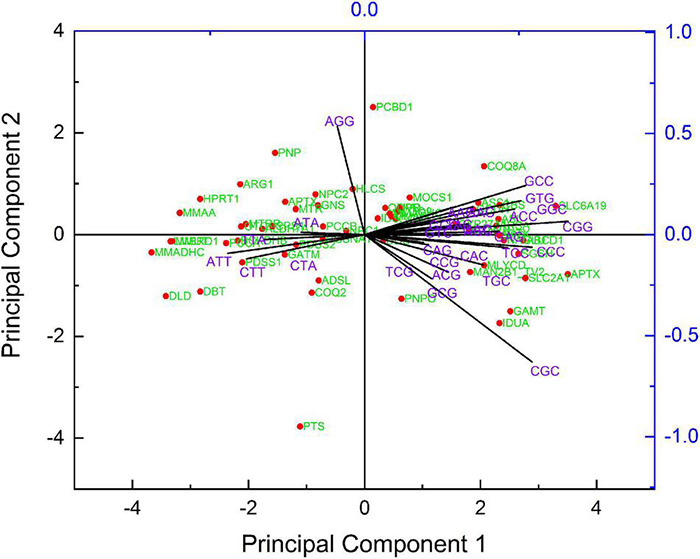
A biplot depiction of PCA. Dots are representing the sequences, whilst arrows are showing codons. Eclipse showing a 95% confidence limit.

The biplot arrows indicate the preferred codons from each sequence. The farthest vector (codon) has the maximum effect on PC. The eclipse enclosed the sequences with 95% confidence based on PCA on biplots (Figure 9). The dots are the PCA score of the sequences. *PCBD1* and *PTS* genes were present outside of 95% confidence eclipse. The scree plot revealed that PC1 captured 51.56% of the variation, and PC2 captured 6.13%.

## Discussion

In the present study, the codon usage pattern was analyzed for 183 transcripts belonging to 60 genes involved in neurodegeneration with metabolic disturbances. Initial overall nucleotide composition analysis revealed a random pattern for A, T, C, and G nucleotide usage in the sequences. Variation was observed at total nucleotide composition as well as GC composition. The nucleotide component C had a maximum range of variability of 22.35%, while nucleotide G had the least (14.34%). The GC3 component showed the most variation of all positions, possibly due to the degeneracy at the third codon position.

Dinucleotide frequency significantly influences codon usage bias and can be considered a genetic signature for a species (De Amicis and Marchetti, 2000). Underrepresentation of the TpA dinucleotide has been reported in multiple studies. As TpA is more susceptible to degradation by cellular RNases, owing to its mRNA destabilizing effect, contribution to stop codons (TAA and TAG) (Khandia et al., 2019), and selectional forces that tend to keep the TpA content low. CpG dinucleotides are also predisposed to mutations by deamination of 5-methylcytosine at CpG sites resulting in C?T changes. The dinucleotide CpG is approximately 42 times more mutable than predicted from random mutation; however, the exact proportion cannot be estimated due to the variable degree of methylation of cytosine in vertebrates (Cooper and Youssoufian, 1988). For this reason, the expression constructs for protein expression for protein production and gene therapy are designed in a way to avoid CpG (Bauer et al., 2010).

Despite selection forces acting against the dinucleotide pair TpA and CpG, our study found an unbiased representation of the TpA and CpG dinucleotides in 18.33 and 23.33% of sequences, respectively. In a study related to a humanized green fluorescent protein, consideration was given to 60 CpG residues within the coding region. Expression of a detectable amount of protein was decreased with decreasing number of CpG and was independent of the promoter used. A similar experiment reported that CpG depleted mRNA was decreased fivefold in the nucleus and eightfold in the cytoplasm. A decrease in the GFP reporter expression associated with CpG depletion was more related to a decline in the copy number of mRNA than translational efficiency, and the effect was gene-independent (Bauer et al., 2010). This result indicates that intragenic CpG influences *de novo* transcriptional activity. Such experimental evidence implies that although CpG tends to mutate faster than other nucleotide combinations and makes the gene vulnerable to loss of function, it is still essential for optimal gene expression; hence, a fine-tuned balance is needed to achieve optimal protein expression. Apart from *de novo* transcription, CpG has a role in the stability of RNA transcripts, and with an increasing proportion of CpG, mRNA stability and subsequent protein expression also increase (Duan and Antezana, 2003).

It is evident that all the genes related to metabolism or metabolite transport need to be highly expressed in the cells to meet metabolism and metabolite transport demands. Despite their tendency towards mutation and loss of function, the genes tended to retain CpG at an adequate level, explaining well the unbiased usage of CpG in our study and underscoring the selectional forces that keep the CpG at a certain level to maintain high expression.

A *BRCA1* or *BRCA2* mutant chicken DT40 cell line model for spontaneous mutation exhibited an 11 times higher likelihood of NCG to NTG mutation relative to the mean mutation rate (Zámborszky et al., 2017). In our study, we found strikingly high RSCU for CTG (RSCU > 1.6; highest 4.73) and GTG (RSCU > 1.6; highest 3.28) codons in some genes with over-representation of CTG and GTG in 78.33 and 68.33% of genes, respectively. This finding correlated well with the transition of CpG dinucleotide to TpG. It is further strengthened by the fact that the predecessors of CTG and GTG (codons CCG and GCG) were over exhibited only in 3.26 and 4.34% of coding sequences but underrepresented for both CCG and GCG in 85.86 and 80.97% of genes. Thus, underrepresentation can be attributed to the conversion of CCG and GCG to CTG and GTG, culminating in CTG and GTG overrepresentation.

Several factors affect the biased codon choices, including genetic drift, mutation pressure, natural selection, composition, secondary protein motifs, protein’s physical properties, transcriptional factors, and external environment, tRNA abundance etc. (Ikemura, 1981). However, natural selection, mutation pressure with genetic drift (Chen et al., 2014; LaBella et al., 2019), and compositional constraints (Jia et al., 2015) are major factors. In addition, various analyses like parity, neutrality, ENc-GC3 analysis, and abundance of specific codons and dinucleotides suggest the presence of selection as a significant force and mutation force. Investigation of the role of compositional properties on codon bias revealed that three out of four nucleotides at the second position of the codon significantly impact the bias (A2, C2, and T2). The A2 nucleotides negatively correlated with Nc, while C2 and T2 were positively correlated. This result could be explained by Saier (2019) work, who explained that the second nucleotide position is the most important in determining the nature of the genetic code. Overall based on our analyses, it can be inferred that selection, mutation and composition are the forces that might be responsible for shaping codon usage. In living organisms, the GC content ranges from approximately 20% GC to 80% GC. Upon plotting the GC content variation at three codon positions against overall GC content, there appeared a positive correlation between GC content in a codon with a total GC content of the genome; however, the steepness of the slope differed with a rank of third, first, and second codon positions (Muto and Osawa, 1987). Since the mutations are random, the advantageous one will be selected. The constraints affecting the mutation are highest on codon position two, while least on codon position three. This observation can be attributed to the fact that the second position of the codon specifies the type of amino acid, while the first one specifies a specific amino acid. The third position is redundant since several bases specify an amino acid. How position two of the codon specifies the type of amino acid can be understood by the example of when T, A, and C are present at the second position: all resulting amino acids are hydrophobic, hydrophilic, and semipolar. The only exception is G; when it is present at the second position, similar to C at the second position, it results in semipolar amino acids with two exceptions (arginine, a strongly hydrophilic amino acid, and UGA, a stop codon).

A regression analysis between the Nc with the nucleotide content present at the third position of the codon revealed a positive association with %A3 and %T3 and a negative correlation with %C3 and %G3. The %T3 had the highest regression coefficient and a positive correlation. Parity plot analysis revealed that T was preferred over A, and G is preferred over C. The disproportionate usage of these nucleotides suggests the natural selection of codon usage bias of genes (Uddin and Chakraborty, 2016) linked to neurodegeneration. Neutrality analysis indicates the dominance of selection and other forces, such as compositional, in shaping codon usage. Mutational force only contributed 17.32 and 31.5% variance in GC12 was attributed to GC3. Similar to Nc, ICDI is also a parameter to evaluate the codon usage bias, and its value ranges between 0 and 1. Higher ICDI values (toward 1) indicate the highest codon usage bias. In the present study, the average ICDI value was 0.182 ± 0.008, indicating a generally low bias.

Nc analysis revealed a relatively low bias in codon usage. This finding was coupled with the fact that these genes were highly expressed with high CAI values. The CAI value quantifies the synonymous codon usage bias for a DNA or RNA sequence (and the codon usage similarities between the gene and a reference set). High CAI suggests a very high selectional force on a gene to selectively use a codon contributing to high-level protein expression (Sharp and Li, 1987; Puigbò et al., 2007). The genes with higher CAI values tend to utilize more optimal codons. The CAI value varied between 0.885 and 0.71. In *E*. *coli*, the highest CAI (0.84) has been reported for the *rplL* gene encoding ribosomal protein L7/12, one of the most abundant proteins present in the species (DiRienzo and Inouye, 1979). In the present study, all the genes had high CAI values, indicating higher expression of genes and the importance of these genes in physiological functions. Upon regression the CAI values to the nucleotide composition present at the third position of the codon, a very low regression coefficient indicated that mutational forces were not affected by the gene expression and expression was driven mainly by selectional forces.

## Conclusion

The present study explored the codon usage pattern and various forces applied on 183 transcripts of 60 genes involved in neurodegeneration associated with metabolic ailments. Analyses revealed a random pattern of the overall composition of the four standard nucleotides, and nucleotide C had a maximum range of variability of 22.35% in terms of total nucleotide components. Across the protein length, up to 800 amino acids, with increasing length, GC12 remained constant while GC3 fluctuated widely. The overall codon usage bias was low with higher Nc values and low ICDI. An investigation into the effects of compositional parameters on codon usage bias revealed that the second position of a codon is critical, as determined by a significant correlation of A2, C2, and T2 with Nc (*p* > 0.001). The genes were highly expressed, evidenced by their very high CAI values. This higher expression shows their involvement in critical physiological processes. Other parameters such as neutrality analysis, parity plot, and Nc-GC3 curve indicated the dominance of selection pressure along with the presence of compositional and mutational constraints. The transcripts exhibited under-representation of dinucleotides TpA and CpG due to selectional pressure. However, unbiased representation (odds ratio > 0.78) of TpA and CpG dinucleotides was observed in 18.33 and 23.33% of genes. These dinucleotides are important as part of regulatory elements (TATA box, stop codons, polyadenylation signal) in the thermodynamic stability of mRNA and missense mutations. The unbiased representation of these dinucleotides suggests selectional forces finely tune the CpG content level to obtain the optimum rate of protein expression for the high demand of these metabolism and metabolite transfer-related genes. The loss of this fine-tuning leads to neurological ailments. Notably, we observed very high RSCU values for CTG and GTG codons resulting from the transition of C to T. This observation indicates the mutational forces move forward to eliminate the CpG and selection pressure in the reverse direction to maintain high protein expression and this critical balance fine tune the CpG content in genes associated with neurodegeneration.

## Data Availability Statement

The original contributions presented in the study are included in the article/Supplementary Material, further inquiries can be directed to the corresponding author/s.

## Ethics Statement

Ethical review and approval were not required for the study on human participants in accordance with the local legislation and institutional requirements.

## Author Contributions

RK: conceptualization. RK and AS: methodology and writing—original draft preparation. RK, TA, AA, YA, SA, and AMA: validation. RK, TA, and AA: formal analysis. RK, AS, TA, AA, YA, SA, AMA, and MK: writing—review, editing, and visualization. MK: final validation. All authors have read and agreed to the published version.

## Conflict of Interest

The authors declare that the research was conducted in the absence of any commercial or financial relationships that could be construed as a potential conflict of interest.

## Publisher’s Note

All claims expressed in this article are solely those of the authors and do not necessarily represent those of their affiliated organizations, or those of the publisher, the editors and the reviewers. Any product that may be evaluated in this article, or claim that may be made by its manufacturer, is not guaranteed or endorsed by the publisher.

## Footnotes

1
https://www.dnastar.com/software/lasergene/

2
www.kazusa.or.jp

## Supplementary Material

Supplementary Table 1 Nucleotide composition and composition at various codon positions for genes involved in neurodegeneration.
